# Unravelling upright events: a descriptive epidemiology of the behavioural composition and temporal distribution of upright events in participants from the 1970 British Cohort Study

**DOI:** 10.1186/s12889-024-17976-2

**Published:** 2024-02-21

**Authors:** Joshua Culverhouse, Melvyn Hillsdon, Richard Pulsford

**Affiliations:** Department of Public Health and Sport Sciences, University of Exeter, Richard’s Building, St Luke’s Campus, Heavitree Road, Exeter, EX1 2LU UK

**Keywords:** Sitting interruptions, Sedentary breaks, Accelerometry, Middle aged, Standing, Stepping, Postures

## Abstract

**Background:**

Continued proliferation of accelerometers in physical activity research has opened new avenues for understanding activity behaviours beyond simple aggregate measures of frequency and duration. This study explores the standing and stepping composition, and the temporal distribution, of upright events, and investigates their associations with sociodemographic and health factors.

**Methods:**

Participants from the 1970 British Cohort Study wore activPAL3 accelerometers for seven days. Event-based analysis was used to extract a time series of upright, standing, and stepping events. Derived metrics included daily number of upright and stepping events, total upright and stepping time, the burstiness of upright events and burstiness of sedentary events (burstiness refers to the pattern of how physical activity and sedentary behaviour are distributed throughout a given time period), within-event stepping proportion, within-event step count, and stepping cadence. Generalized linear regression models, adjusted for total step count, were employed to explore associations between derived metrics and sociodemographic and health-related factors.

**Results:**

A total of 4527 participants, provided 30992 valid days (≥ 10 h of waking wear) and 1.64 million upright events. Upright event composition and temporal distribution varied across a range of sociodemographic and health-related factors. Females had more upright events than males (4.39 [3.41,5.38] n), spent more time upright, and exhibited burstier patterns of upright events (0.05 [0.04,0.05] *B*_n_). Individuals with higher BMI had fewer upright events and a lower daily step count, but their temporal distribution of upright events was less bursty (overweight -0.02 [-0.02,-0.01] *B*_n_; obese -0.03 [-0.04,-0.02] *B*_n_), and upright events had a higher step count. People in active occupations were upright for longer, displayed burstier patterns of upright events (standing 0.04 [0.03,0.05] *B*_n_; physical work 0.05 [0.04,0.05] *B*_n_; heavy manual 0.06 [0.04,0.07] *B*_n_), with more variable durations and shorter, slower paced stepping events compared with sedentary occupations.

**Conclusions:**

This study has revealed novel phenotypes of standing and sitting that go beyond simple aggregate measures of total steps, step event duration or time between events. People with the same volume of stepping and frequency of gaps between upright events can accumulate their steps in very different ways. These differences and associations with population sub-groups, which persisted after adjustment for total stepping volume, may have important relations with functional and health outcomes. The findings lay the groundwork for future studies to investigate how different sitting and standing phenotypes can add to our understanding of the relationship between physical activity and health.

**Supplementary Information:**

The online version contains supplementary material available at 10.1186/s12889-024-17976-2.

## Introduction

The proliferation of the use of accelerometer-based measures in physical activity research has permitted new insights into physical activity behaviour that were not previously possible with self-report measures [1]. The availability of continuous 24-h monitoring and high resolution, time stamped movement data mean it is now possible to advance beyond reporting average daily and weekly volumes of physical activity to investigate how people accumulate a given volume of movement over time [2, 3]. With the high accuracy of postural classification using thigh worn devices [4], time stamped data on postural behaviours makes it possible to extract an event-based time-series of contiguous upright and sedentary (sitting/reclining) events for each 24 h period of observation [5]. Consequently, event-based analyses can be used to investigate the frequency, duration, and intensity of upright events (also termed sitting interruptions or sedentary breaks) [6]. In addition, the temporal distribution of events can be assessed including how clustered events are [7], how transient or sustained they are [8], and the time of day they take place [9]. Although most physical activity research using accelerometers is still restricted to a small number of aggregate metrics, such as the number of minutes of at least moderate intensity activity, or time spent sedentary, there is growing research interest in utilising time stamped data to move beyond these simple metrics [10]. For example, frequency of postural (sit-to-stand) transitions has been associated with metabolic health [11, 12]; the time of day when physical activity is undertaken has been associated with cardiovascular disease risk and mortality [2, 13]; and how fragmented (transient) or sustained physical activity events are has been associated with a range of age related health outcomes [14–17].

When in an upright posture people can be either standing or ambulating, with evidence that stepping confers greater metabolic health benefits than standing-only upright events [11]. Therefore, the next step beyond counting the frequency of upright events is to characterise their durations, temporal distribution, and their composition (the mix of standing and stepping). Standing and stepping events within each upright event can further be characterised by their frequency, duration, and stepping rate (cadence) [6]. A recent cross-sectional study, examined the associations between sitting interruptions (upright events), demographic factors, diabetes status, and BMI [18]. The frequency of all interruptions, active interruptions (≥ 5 min duration and/or ≥ 2 min stepping) and ambulatory interruptions (≥ 2 min stepping) were extracted from 7-days of thigh worn activPAL data. Fewer interruptions of any type and fewer steps per day were associated with higher BMI and diabetes status. However, the study did not take account of the stepping vs standing composition of upright events, the temporal distribution of events, the number and composition of stepping events, and did not control for all steps accumulated. This is important as the proportions and total duration of standing and stepping, the number and distribution of stepping and standing events, and the stepping volume and cadence can all vary even when the total number and duration of upright events is the same. Moreover, the temporal distribution of upright events can vary while the frequency, duration and composition of events is the same. These features of activity accumulation, all have the potential to be associated with health outcomes and warrant further investigation.

Given emerging evidence regarding the importance of the patterns in which physical activity is accumulated [19], a deeper understanding of the composition, and temporal distribution, of upright events, may provide new insights into their relationship with health outcomes and how they differ between people. Such, insights may be masked when behaviours such as sitting, standing, and stepping are confined to measures of frequency, average duration, average time between events, or the volume of time in each event over different observation periods. To our knowledge, no study to date has fully described the composition and temporal distribution of upright events recorded in a free-living setting. Therefore, in this study we address this need by providing a comprehensive description of the composition and temporal distribution of free-living uprights events and how they vary by demographic and health factors, in a cohort of middle-aged UK adults.

## Methods

### Study design

This study used data from the 1970 British Cohort Study (BCS70) [20]. The participant profile of the BCS70 is detailed elsewhere [21]. Briefly, participants were born during a single week in 1970 across England, Scotland, and Wales, and have been followed up through childhood and adulthood. For the 2016–2018 assessment, when participants reached 46 years of age, a comprehensive interview and nurse visit were conducted, along with the invitation to wear an accelerometer, which was attached during the visit (and returned via post). Participants of BCS70 provided consent, and the cohort study received full ethical approval from the NRES Committee South East Coast-Brighton and Sussex.

### Upright event measurement

Posture and movement behaviour were monitored using the activPAL3 micro (PAL Technologies Ltd., Glasgow, UK), programmed to sample at the default frequency of 20 Hz. The device was waterproofed and attached on the midline anterior aspect of the upper thigh by a trained nurse. Following a previously adopted protocol [22], participants were instructed to wear the device continuously for 7 days (from the day after attachment), 24 h·d^−1^. The raw.datx files were processed using PALbatch software v.8 to produce the stepping bouts.csv output. The proprietary CREA v.1.3 classification algorithm was used with 24-h non-wear protocol and default recommended minimum durations of 10 s for upright and sedentary (non-upright) periods. Upright events were output as a time series with a date and time stamp for each event. Upright events were defined as the time between two consecutive transitions from a sedentary posture to an upright posture, and the subsequent transition from an upright posture back to a sedentary posture.

#### Data cleaning

CREA-classified non-wear periods and any data after a participant’s first non-wear bout were removed, as participants were instructed not to reattach the device during wear. To isolate valid waking wear time from sleep, waking wear time was estimated using the first upright event ≥ 10 s after 03:00 h until the event preceding the one that crossed the following midnight. This estimation method was based on the average midsleep point reported in a large UK cohort study [23], and assumed that the next upright event ≥ 10 s after this midsleep point represented the arise time. The first day of recording was a partial day and was excluded. A minimum of 10 h of waking wear and > 3 upright events (≥ 10 s) was required for a day to be valid, and inclusion criteria for this study was a minimum of six valid days. Physical activity metrics (described below) were derived for the waking wear time of each 24-h period.

#### Daily summary metrics

The mean daily number of upright and stepping events (n), and total duration of upright time, standing time, and stepping time (h·d^−1^) were derived per person.

#### Composition of upright events

The composition of each individual upright event was characterised by its duration (mins), proportion of time stepping (%), number of stepping events (n), and step count (steps). The per person mean of these metrics were used for analysis.

#### Stepping metrics

The mean daily step count, mean duration of each stepping event (min), mean number of steps per stepping event (steps), and the mean step-weighted cadence of all stepping events (steps·min^−1^) was derived per person. A ten-step minimum was used when calculating cadence (except when deriving daily step count) as 6–10 consecutive steps have been deemed the minimum required to accurately record stepping cadence [24]. Step-weighting the average gave more importance to longer stepping events and reduced the influence of shorter step events with higher cadences that were less likely to represent purposeful walking. Finally, the mean daily step count of all steps recorded was derived and used as the measure of physical activity (stepping) volume.

#### Temporal distribution of events

The temporal distribution of events was defined by the ‘burstiness’ parameter. Burstiness is based on the variation in inter-event times and uses the following equation, which corrects for the number of events [7]:

$${B}_{n}=\frac{\sqrt{n+1} \left(\frac{\sigma }{\langle \tau \rangle }\right) - \sqrt{{\text{n}}-1}}{\left(\sqrt{n+1}-2\right) \left(\frac{\sigma }{\langle \tau \rangle }\right)+ \sqrt{{\text{n}}-1}}$$

where *n*, σ, and 〈τ〉 denote the number of events, the standard deviation inter-event time, and the mean of inter-event time, respectively. Burstiness was calculated per day and averaged per person. The burstiness parameter can take a value between -1 to + 1, with high values indicating higher burstiness, which is a larger standard deviation of the inter-event times compared to the mean. Low burstiness would reflect a relatively consistent distribution of events across the day, whereas high burstiness would reflect more clustering of events at points in the day, followed by longer gapes between these clusters. The same equation was applied to both upright and sedentary events to provide both upright event burstiness and sedentary event burstiness metrics.

**Fig. 1:**
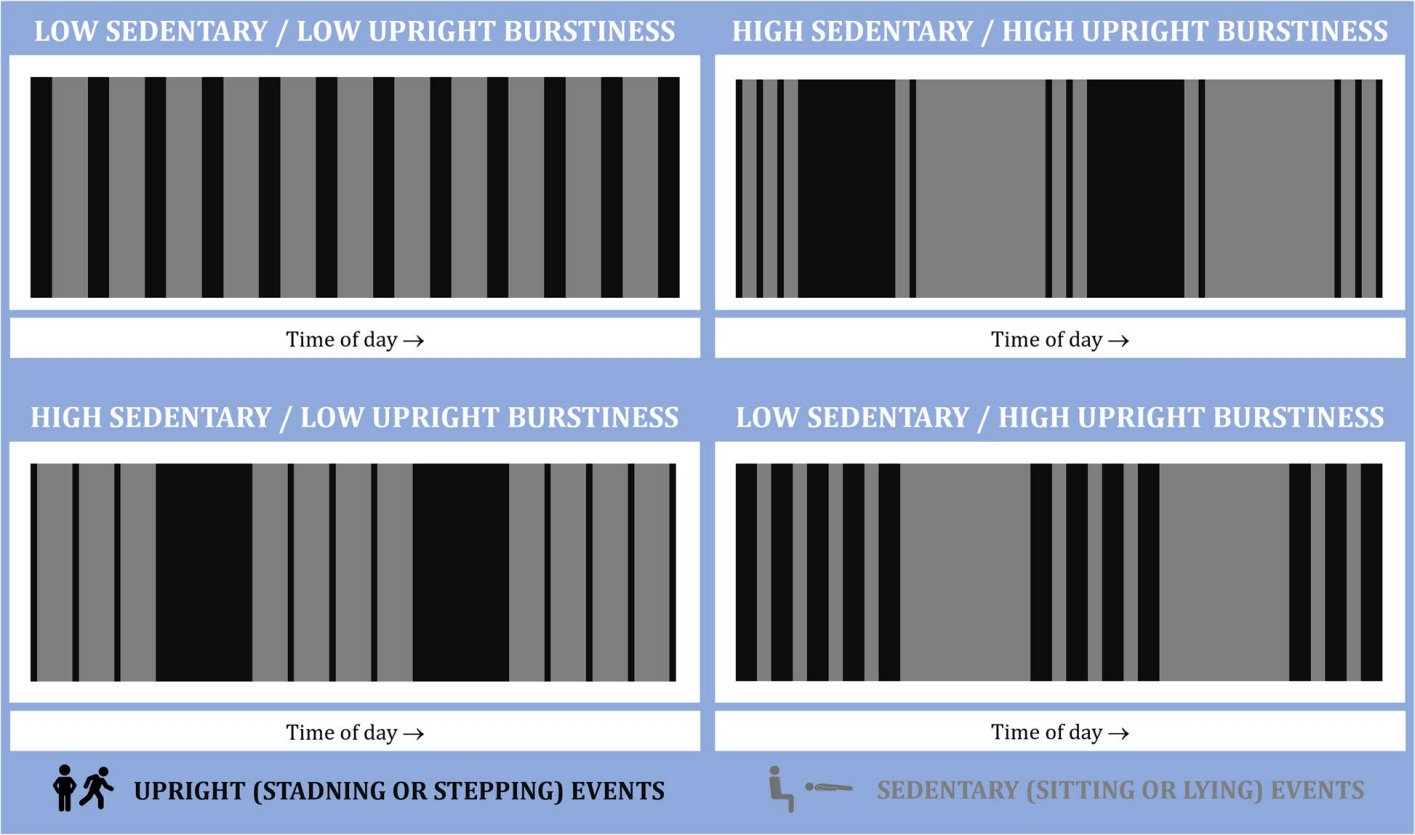
Diagrammatic examples of burstiness for sedentary and upright events. Legend: The examples are matched for daily event count, waking wear time, and duration of upright events to ensure a fair comparison. Low burstiness is represented by a coefficient of -1, while high burstiness is indicated by a coefficient of + 0.5

The illustration in Fig. 1 showcases both high and low burstiness for sedentary and upright events. The low/low example displays an even distribution of both event types throughout the day. The high sedentary / low upright example exhibits consistent sedentary event durations but features two longer upright events amid several shorter ones, achieving high burstiness in sedentary events through a mix of durations. Conversely, high burstiness in upright events (low sedentary / high burstiness) is characterized by clusters of short gaps between upright events, followed by more extended sedentary periods. The high/high example demonstrates a combination of both scenarios. These examples visually elucidate burstiness, although real-world movement data presents a more intricate and diverse picture.

### Demographic and health-related characteristics

Participants provided information on a range of socio-demographic, lifestyle, and health factors. Body Mass Index (BMI in KG.m^2^) was calculated for nurse measured height (portable Leicester stadiometer) and weight (Tanita BF—522W scales), and categorised as under-weight (< 18.5), normal-weight (18.5–24.9), overweight (25.0–25.9), obese (30.0–34.9), or morbidly obese (≥ 35.0). Educational qualification was reported and classified into the following: none, GSCE, A-level, degree. Socio-economic status was reported using the five-class National Statistics Socio-economic Classification (NS-SEC) [25], which categorizes occupations hierarchically ranging from high-level managerial/professional roles to routine jobs. The European Union Statistics on Income and Living Conditions (EU-SILC) [26] provided disability categorisation ranging from: none, some extent, severely hampered. Occupational activity was classified into the following: sitting, standing, physical work, and heavy manual work. Self-reported smoking status was grouped into four categories: never, past smoker, occasional smoker, daily smoke. Self-rated health was categorized as poor, fair, good, very good, or excellent, and was used here as a simple measure of general health.

### Statistical analyses

Participants with six or more valid days of activPAL wear (≥ 10 waking wear hours) and complete demographic and health-related data were included in the analyses. Generalized linear regression models were employed to describe and compare upright event metrics across sex, socio-economic status, education level, disability status, BMI classification, smoking status, and self-rated health; additionally adjusted for waking wear time and mean daily step count. Multi-collinearity was checked using the variance inflation factor (VIF). All statistical analyses were performed using Stata v17.0 (StataCorp, USA).

#### Sensitivity analyses

To assess the robustness of our results, analyses were repeated to assess the impact of EU-SILC disability classification in the analytical sample. These included rerunning analyses excluding participants classified as severely hampered, and again excluding the ‘some extent’ and severely hampered classifications.

## Results

### Participant characteristics

A total of 4526 participants (78% of the 5795 activPAL files available) had six or more valid days (≥ 10 h·d^−1^ waking wear and > 3 upright events) of activPAL data. This resulted in 30,992 valid days with an average waking wear time of 16.2 ± 0.9 h·d^−1^ (mean ± SD), and a total of 1,638,009 upright events. Participants had an average of 52.9 ± 15.3 upright events per day, and 198.4 ± 69.6 stepping events per day. Upright duration averaged 6.4 ± 1.9 h·d^−1^, with stepping duration 2.0 ± 0.7 h·d^−1^, and the mean daily step count for was 9389 ± 3586 steps·d^−1^. Table 1 provides a descriptive summary of upright events by sex for this sample. A total of 3965 participants had valid accelerometer wear and complete covariates data, this sample was included in regression analyses. Demographics are presented in Table 1. For all regression models, VIF was < 2 for each independent variable.

**Table 1:** Descritpives of participant demographics (n (%)), and device-derived metrics (mean(SD))

**Demographics**	**Men (*n* = 1897)**	**Women (*n* = 2068)**
Highest qualification		
None	523 (56.3%)	406 (43.7%)
GCSE	571 (46.4%)	660 (53.6%)
FE	252 (40.1%)	377 (59.9%)
HE	551 (46.9%)	625 (53.1%)
Disability		
None	1701 (49.0%)	1771 (51.0%)
Some extent	151 (38.9%)	237 (61.1%)
Severely hampered	44 (42.7%)	59 (57.3%)
Self-rated health		
Excellent	353 (43.4%)	461 (56.6%)
Very good	743 (47.6%)	817 (52.4%)
Good	557 (50.6%)	543 (49.4%)
Fair	211 (51.2%)	201 (48.8%)
Poor	33 (41.8%)	46 (58.2%)
NS-SEC group		
Professional	1049 (52.6%)	947 (47.4%)
Intermediate	564 (46.7%)	643 (53.3%)
Routine	235 (43.5%)	305 (56.5%)
BMI		
Normal (18.5 < 25)	428 (34.8%)	801 (65.2%)
Overweight (25 < 35)	862 (56.8%)	656 (43.2%)
Obese (30 < 35)	540 (52.3%)	493 (47.7%)
Morbidly obese (≥ 35)	27 (25.2%)	80 (74.8%)
Underweight (< 18.5)	40 (51.3%)	38 (48.7%)
Occupational activity		
Sitting	1029 (47.0%)	1159 (53.0%)
Standing	190 (30.3%)	437 (69.7%)
Physical work	510 (52.8%)	455 (47.2%)
Heavy manual	168 (90.8%)	17 (9.2%)
Smoking habits		
Never	954 (58.6%)	1082 (41.4%)
Past smoker	612 (47.6%)	673 (52.4%)
Occasional smoker	93 (51.4%)	88 (48.6%)
Daily smoker	238 (51.4%)	225 (48.6%)
**Device-derived metrics**	**Men (*n* = 2077)**	**Women (*n* = 2387)**
Summary metrics		
Upright events (n)	50.8 (15.5)	54.7 (14.8)
Stepping events (n)	194.7 (72.6)	201.7 (66.8)
Upright duration (h)	6.3 (1.9)	6.6 (1.9)
Standing duration (h)	4.3 (1.5)	4.6 (1.5)
Stepping duration (h)	2.0 (0.7)	2.0 (0.7)
Pattern metrics		
Upright event burstiness (*B*_n_)	0.28 (0.10)	0.31 (0.08)
Sedentary event burstiness (*B*_n_)	0.28 (0.09)	0.27 (0.08)
Stepping metrics		
Step count (steps)	9451 (3670)	9334 (3483)
Step-weighted cadence (steps/min)	88.8 (9.2)	90.1 (8.4)
Stepping event duration (s)	32.5 (9.2)	29.7 (7.5)
Step count per stepping event (steps)	46.1 (17.8)	42.4 (14.4)
Composition metrics		
Upright event duration (min)	8.0 (3.7)	7.7 (3.8)
Stepping proportion (%)	35.8 (6.4)	35.5 (5.9)
Stepping events per upright event (n)	9.1 (4.1)	8.9 (3.6)
Step count per upright event (n)	198.8 (97.5)	179.6 (79.4)

*n* Number/count, *h* Hour, *min* Minute, *s* Seconds

### Characterisation, composition, and temporal distribution of upright events according to sociodemographic and health factors

All analyses were adjusted for average number of steps per day, therefore, the reported variances across demographics for these metrics were present when adjusting for a proxy measure of volume. Females moved to an upright posture more frequently than males (4.39 [3.41,5.38] n), spent more time upright and standing, and the upright events were more bursty than males (more clustered together with longer between event times (0.05 [0.04,0.05] *B*_n_)) (Table 2). Although there was no difference in the total number of steps taken between the sexes, females recorded a higher daily frequency of stepping events (13.39 [10.24,16.53] n), with shorter durations; with fewer steps per stepping and upright event, but steps taken were at a higher average cadence (0.89 [0.39,1.40] steps·min^−1^) (Tables 3 and 4).

**Table 2:** Daily summaries metrics by socioeconomic and health-related factors in adults aged-46 (BCS70)

		**Upright events (n)**		**Upright duration (min)**		**Standing duration (min)**		**Stepping duration (min)**		**Upright event burstiness (*B***_**n**_**)**		**Sedentary event burstiness (*B***_**n**_**)**	
	**N**	**B**	**[95% CI]**	**B**	**[95% CI]**	**B**	**[95% CI]**	**B**	**[95% CI]**	**B**	**[95% CI]**	**B**	**[95% CI]**
**Sex** (Ref: Male)	1897	Ref		Ref		Ref		Ref		Ref		Ref	
Female	2068	4.39^***^	[3.41,5.38]	22.68^***^	[17.20,28.16]	22.86^***^	[17.69,28.03]	-0.18	[-0.91,0.54]	0.05^***^	[0.04,0.05]	0.00	[-0.01,0.00]
**Highest qualification** (Ref: None)	929	Ref		Ref		Ref		Ref		Ref		Ref	
GCSE	1231	0.22	[-1.03,1.46]	0.85	[-6.11,7.81]	0.59	[-5.97,7.15]	0.26	[-0.66,1.18]	0.00	[-0.00,0.01]	-0.01	[-0.01,0.00]
FE	629	0.10	[-1.41,1.60]	0.97	[-7.45,9.38]	0.53	[-7.41,8.46]	0.44	[-0.67,1.55]	0.00	[-0.00,0.01]	-0.01^*^	[-0.02,-0.00]
HE	1176	-0.95	[-2.35,0.45]	-6.87	[-14.62,0.87]	-6.45	[-13.76,0.85]	-0.42	[-1.45,0.60]	0.00	[-0.01,0.01]	-0.02^***^	[-0.03,-0.01]
**Disability** (Ref: None)	3472	Ref		Ref		Ref		Ref		Ref		Ref	
Some extent	388	-0.48	[-2.12,1.15]	3.57	[-5.43,12.56]	3.29	[-5.19,11.78]	0.27	[-0.92,1.46]	0.00	[-0.01,0.01]	0.00	[-0.01,0.01]
Severely hampered	103	-2.72	[-6.00,0.56]	-17.26	[-34.89,0.37]	-17.69^*^	[-34.32,-1.07]	0.44	[-1.90,2.77]	0.01	[-0.01,0.03]	-0.02	[-0.04,0.00]
**Self-rated health** (Ref: Excellent)	814	Ref		Ref		Ref		Ref		Ref		Ref	
Very good	1560	0.83	[-0.42,2.08]	9.76^**^	[2.83,16.69]	7.69^*^	[1.16,14.23]	2.07^***^	[1.15,2.98]	0.01^*^	[0.00,0.01]	0.00	[-0.00,0.01]
Good	1100	0.94	[-0.45,2.32]	8.50^*^	[0.78,16.23]	6.61	[-0.67,13.89]	1.89^***^	[0.87,2.91]	0.01^*^	[0.00,0.02]	0.00	[-0.00,0.01]
Fair	412	0.53	[-1.37,2.42]	11.46^*^	[0.98,21.94]	8.83	[-1.05,18.72]	2.63^***^	[1.24,4.01]	0.01	[-0.00,0.02]	0.00	[-0.01,0.01]
Poor	79	-1.29	[-5.07,2.49]	-4.75	[-25.53,16.04]	-5.73	[-25.33,13.87]	0.98	[-1.77,3.73]	-0.01	[-0.04,0.01]	-0.01	[-0.04,0.01]
**NS-SEC group** (Ref: Professional)	1996	Ref		Ref		Ref		Ref		Ref		Ref	
Intermediate	1207	1.81^**^	[0.68,2.94]	13.16^***^	[6.75,19.57]	10.78^***^	[4.73,16.82]	2.38^***^	[1.54,3.23]	0.01	[-0.00,0.01]	0.01	[-0.00,0.01]
Routine	540	-0.54	[-2.09,1.02]	6.86	[-1.96,15.67]	4.57	[-3.74,12.87]	2.29^***^	[1.12,3.46]	0.00	[-0.01,0.01]	0.01^*^	[0.00,0.02]
**Body mass index** (Ref: 18.5 < 25)	1229	Ref		Ref		Ref		Ref		Ref		Ref	
Overweight (25 < 35)	1518	-3.18^***^	[-4.30,-2.06]	-6.84^*^	[-13.09,-0.60]	-6.38^*^	[-12.27,-0.49]	-0.47	[-1.29,0.36]	-0.02^***^	[-0.02,-0.01]	0.00	[-0.01,0.01]
Obese (30 < 35)	1033	-6.38^***^	[-7.65,-5.12]	-7.09^*^	[-14.13,-0.06]	-6.40	[-13.03,0.24]	-0.69	[-1.63,0.24]	-0.03^***^	[-0.04,-0.02]	0.00	[-0.01,0.01]
Morbidly obese (≥ 35)	107	-12.97^***^	[-15.87,-10.06]	-3.88	[-20.22,12.47]	-2.43	[-17.84,12.98]	-1.44	[-3.61,0.72]	-0.04^***^	[-0.06,-0.02]	0.01	[-0.01,0.03]
Underweight (< 18.5)	78	-8.15^***^	[-11.52,-4.78]	-8.04	[-26.67,10.58]	-7.59	[-25.15,9.97]	-0.45	[-2.92,2.01]	-0.01	[-0.03,0.01]	0.01	[-0.01,0.03]
**Occupational activity** (Ref: Sitting)	2188	Ref		Ref		Ref		Ref		Ref		Ref	
Standing	627	-0.39	[-1.73,0.96]	69.24^***^	[61.84,76.65]	63.28^***^	[56.30,70.27]	5.96^***^	[4.98,6.94]	0.04^***^	[0.03,0.05]	0.02^***^	[0.01,0.02]
Physical work	965	-0.2	[-1.45,1.05]	62.74^***^	[55.80,69.68]	54.16^***^	[47.61,60.70]	8.58^***^	[7.66,9.50]	0.05^***^	[0.04,0.05]	0.02^***^	[0.01,0.03]
Heavy manual	185	-0.27	[-2.60,2.06]	75.74^***^	[62.53,88.94]	61.31^***^	[48.85,73.76]	14.43^***^	[12.69,16.18]	0.06^***^	[0.04,0.07]	0.03^***^	[0.01,0.04]
**Smoking habits** (Ref: Never)	2036	Ref		Ref		Ref		Ref		Ref		Ref	
Past smoker	1285	1.52^**^	[0.50,2.54]	-5.65	[-11.33,0.02]	-6.27^*^	[-11.63,-0.92]	0.62	[-0.13,1.37]	0.00	[-0.01,0.00]	0.00	[-0.01,0.00]
Occasional smoker	181	2.64^*^	[0.44,4.85]	15.4^*^	[3.12,27.73]	15.23^*^	[3.63,26.83]	0.19	[-1.43,1.82]	0.00	[-0.01,0.01]	0.00	[-0.02,0.01]
Daily smoker	463	2.79^***^	[1.28,4.30]	11.82^**^	[3.37,20.27]	10.66^**^	[2.69,18.62]	1.16^*^	[0.04,2.28]	-0.01^*^	[-0.02,-0.00]	-0.01^*^	[-0.02,-0.00]

Multivariate linear regressions of upright event metrics. Presented as the unstandardised regression coefficient (B) and 95% confidence intervals [95% CI]. Mutually adjusted for all socioeconomic, lifestyle and health factors, and daily wear time. Additionally adjusted for daily step count

*N* Sub-group sample size

^*^p < 0.05, ^**^p < 0.01, ^***^p < 0.001

**Table 3:** Stepping metrics by socioeconomic and health-related factors in adults aged-46 (BCS70)

		**Daily steps (steps)**		**Step-weighted cadence (steps·min**^**−1**^**)**		**Stepping events (n)**		**Duration of stepping events (s)**		**Steps per stepping event (n)**	
	**N**	**B**	**[95% CI]**	**B**	**[95% CI]**	**B**	**[95% CI]**	**B**	**[95% CI]**	**B**	**[95% CI]**
**Sex** (Ref: Male)	1897	Ref		Ref		Ref		Ref		Ref	
Female	2068	-26.89	[-251.00,197.21]	0.89^***^	[0.39,1.40]	13.39^***^	[10.24,16.53]	-3.16^***^	[-3.63,-2.69]	-4.46^***^	[-5.37,-3.54]
**Highest qualification** (Ref: None)	929	Ref		Ref		Ref		Ref		Ref	
GCSE	1231	-216.65	[-501.06,67.77]	-0.05	[-0.70,0.60]	0.78	[-3.21,4.77]	-0.05	[-0.65,0.55]	-0.25	[-1.40,0.91]
FE	629	-137.28	[-480.97,206.41]	-0.09	[-0.87,0.69]	-1.58	[-6.40,3.24]	0.35	[-0.37,1.07]	0.29	[-1.11,1.68]
HE	1176	343.72^*^	[24.32,663.11]	0.41	[-0.31,1.14]	-8.07^***^	[-12.56,-3.59]	1.42^***^	[0.75,2.10]	2.13^**^	[0.83,3.43]
**Disability** (Ref: None)	3472	Ref		Ref		Ref		Ref		Ref	
Some extent	388	-170.99	[-543.27,201.29]	-0.03	[-0.87,0.82]	-2.95	[-8.17,2.27]	0.26	[-0.52,1.05]	0.42	[-1.09,1.94]
Severely hampered	103	-1270.69^***^	[-2018.05,-523.33]	-1.46	[-3.16,0.23]	-2.47	[-12.97,8.02]	0.49	[-1.08,2.06]	0.43	[-2.62,3.48]
**Self-rated health** (Ref: Excellent)	814	Ref		Ref		Ref		Ref		Ref	
Very good	1560	-713.36^***^	[-996.76,-429.95]	-1.26^***^	[-1.91,-0.62]	9.38^***^	[5.39,13.37]	-1.44^***^	[-2.04,-0.84]	-2.85^***^	[-4.00,-1.69]
Good	1100	-1077.61^***^	[-1391.10,-764.12]	-1.58^***^	[-2.30,-0.86]	11.09^***^	[6.67,15.52]	-1.63^***^	[-2.30,-0.97]	-3.25^***^	[-4.53,-1.96]
Fair	412	-1376.50^***^	[-1807.03,-945.96]	-2.14^***^	[-3.12,-1.15]	14.20^***^	[8.13,20.27]	-1.79^***^	[-2.70,-0.88]	-3.61^***^	[-5.38,-1.85]
Poor	79	-1930.99^***^	[-2791.33,-1070.65]	-2.77^**^	[-4.72,-0.81]	10.21	[-1.89,22.30]	-1.66	[-3.47,0.16]	-3.35	[-6.86,0.16]
**NS-SEC group** (Ref: Professional)	1996	Ref		Ref		Ref		Ref		Ref	
Intermediate	1207	86.3	[-171.06,343.67]	-1.22^***^	[-1.80,-0.64]	6.48^***^	[2.87,10.09]	-0.92^***^	[-1.47,-0.38]	-2.06^***^	[-3.11,-1.01]
Routine	540	522.07^**^	[168.06,876.09]	-1.20^**^	[-2.00,-0.40]	3.24	[-1.73,8.21]	-0.26	[-1.00,0.49]	-1.02	[-2.46,0.42]
**Body mass index** (Ref: 18.5 < 25)	1229	Ref		Ref		Ref		Ref		Ref	
Overweight (25 < 35)	1518	-419.58^**^	[-675.23,-163.93]	-0.20	[-0.78,0.38]	-0.70	[-4.29,2.89]	0.18	[-0.36,0.72]	0.22	[-0.82,1.27]
Obese (30 < 35)	1033	-1232.63^***^	[-1518.66,-946.59]	-0.69^*^	[-1.34,-0.03]	-2.46	[-6.51,1.59]	0.42	[-0.18,1.03]	0.49	[-0.68,1.67]
Morbidly obese (≥ 35)	107	-2546.18^***^	[-3203.49,-1888.87]	-1.75^*^	[-3.25,-0.25]	-8.28	[-17.57,1.01]	1.33	[-0.06,2.73]	1.54	[-1.15,4.24]
Underweight (< 18.5)	78	-1533.72^***^	[-2300.48,-766.96]	-1.28	[-3.03,0.46]	-2.38	[-13.16,8.39]	0.62	[-1.00,2.24]	0.61	[-2.52,3.73]
**Occupational activity** (Ref: Sitting)	2188	Ref		Ref		Ref		Ref		Ref	
Standing	627	1315.56^***^	[1010.91,1620.20]	-3.53^***^	[-4.23,-2.84]	28.57^***^	[24.25,32.88]	-3.92^***^	[-4.57,-3.27]	-7.72^***^	[-8.98,-6.47]
Physical work	965	1701.66^***^	[1421.66,1981.65]	-5.83^***^	[-6.47,-5.18]	35.58^***^	[31.58,39.58]	-4.69^***^	[-5.29,-4.09]	-9.87^***^	[-11.03,-8.71]
Heavy manual	185	2146.26^***^	[1618.79,2673.73]	-8.41^***^	[-9.61,-7.20]	52.40^***^	[44.94,59.86]	-6.34^***^	[-7.45,-5.22]	-13.71^***^	[-15.87,-11.54]
**Smoking habits** (Ref: Never)	2036	Ref		Ref		Ref		Ref		Ref	
Past smoker	1285	123.34	[-109.80,356.48]	-0.28	[-0.81,0.24]	0.00	[-3.27,3.28]	-0.05	[-0.54,0.44]	-0.25	[-1.20,0.70]
Occasional smoker	181	-384.68	[-888.11,118.74]	-0.02	[-1.16,1.12]	4.27	[-2.80,11.33]	-0.78	[-1.84,0.28]	-1.11	[-3.16,0.94]
Daily smoker	463	-908.34^***^	[-1251.84,-564.84]	-1.12^**^	[-1.90,-0.34]	9.14^***^	[4.30,13.97]	-1.15^**^	[-1.87,-0.42]	-2.04^**^	[-3.44,-0.64]

Multivariate linear regressions of upright event metrics. Presented as the unstandardised regression coefficient (B) and 95% confidence intervals [95% CI]. Mutually adjusted for all socioeconomic, lifestyle and health factors, and daily wear time. Additionally adjusted for daily step count (except daily steps)

*N* Sub-group sample size

^*^p < 0.05, ^**^p < 0.01, ^***^p < 0.001

**Table 4:** Upright event composition metrics by socioeconomic and health-related factors in adults aged-46 (BCS70)

		**Duration (min)**		**Stepping proportion (%)**		**Steps (n)**		**Stepping events (n)**	
	**N**	**B**	**[95% CI]**	**B**	**[95% CI]**	**B**	**[95% CI]**	**B**	**[95% CI]**
**Sex** (Ref: Male)	1897	Ref		Ref		Ref		Ref	
Female	2068	-0.24	[-0.49,0.01]	-0.12	[-0.51,0.27]	-17.90^***^	[-22.04,-13.76]	-0.03	[-0.27,0.21]
**Highest qualification** (Ref: None)	929	Ref		Ref		Ref		Ref	
GCSE	1231	-0.13	[-0.45,0.18]	-0.01	[-0.50,0.49]	-3.40	[-8.66,1.85]	-0.07	[-0.37,0.23]
FE	629	-0.14	[-0.52,0.24]	-0.36	[-0.96,0.24]	-3.05	[-9.40,3.30]	-0.19	[-0.55,0.18]
HE	1176	-0.15	[-0.50,0.20]	0.03	[-0.53,0.59]	0.37	[-5.53,6.28]	-0.29	[-0.63,0.04]
**Disability** (Ref: None)	3472	Ref		Ref		Ref		Ref	
Some extent	388	0.30	[-0.11,0.71]	-0.65	[-1.29,0.00]	4.11	[-2.77,10.99]	0.20	[-0.20,0.59]
Severely hampered	103	-0.07	[-0.89,0.76]	0.26	[-1.05,1.56]	7.74	[-6.09,21.57]	0.16	[-0.63,0.95]
**Self-rated health** (Ref: Excellent)	814	Ref		Ref		Ref		Ref	
Very good	1560	0.06	[-0.25,0.37]	0.21	[-0.29,0.70]	-2.84	[-8.09,2.41]	0.28	[-0.02,0.58]
Good	1100	0.02	[-0.33,0.36]	0.34	[-0.21,0.89]	-2.13	[-7.96,3.69]	0.28	[-0.05,0.61]
Fair	412	0.38	[-0.10,0.85]	-0.03	[-0.78,0.72]	-1.05	[-9.05,6.94]	0.54^*^	[0.09,1.00]
Poor	79	-0.19	[-1.15,0.76]	0.93	[-0.57,2.43]	-1.38	[-17.32,14.56]	0.13	[-0.78,1.05]
**NS-SEC group** (Ref: Professional)	1996	Ref		Ref		Ref		Ref	
Intermediate	1207	0.16	[-0.12,0.44]	-0.15	[-0.60,0.30]	-3.75	[-8.50,1.01]	0.37^**^	[0.10,0.64]
Routine	540	0.41^*^	[0.02,0.80]	0.14	[-0.48,0.76]	2.82	[-3.72,9.37]	0.54^**^	[0.16,0.91]
**Body mass index** (Ref: 18.5 < 25)	1229	Ref		Ref		Ref		Ref	
Overweight (25 < 35)	1518	0.31^*^	[0.03,0.59]	0.62^**^	[0.17,1.07]	9.56^***^	[4.83,14.29]	0.25	[-0.02,0.52]
Obese (30 < 35)	1033	0.94^***^	[0.62,1.26]	0.81^**^	[0.31,1.32]	25.06^***^	[19.73,30.40]	0.80^***^	[0.50,1.11]
Morbidly obese (≥ 35)	107	2.37^***^	[1.64,3.10]	1.05	[-0.10,2.20]	48.64^***^	[36.40,60.88]	1.80^***^	[1.10,2.50]
Underweight (< 18.5)	78	1.29^**^	[0.44,2.14]	0.67	[-0.67,2.01]	29.23^***^	[15.03,43.43]	1.16^**^	[0.34,1.97]
**Occupational activity** (Ref: Sitting)	2188	Ref		Ref		Ref		Ref	
Standing	627	1.73^***^	[1.39,2.07]	-1.58^***^	[-2.11,-1.04]	1.31	[-4.37,7.00]	1.90^***^	[1.58,2.23]
Physical work	956	1.42^***^	[1.10,1.73]	-1.38^***^	[-1.87,-0.88]	2.06	[-3.21,7.33]	2.17^***^	[1.87,2.47]
Heavy manual	185	1.83^***^	[1.24,2.42]	-1.09^*^	[-2.01,-0.16]	6.34	[-3.49,16.17]	3.13^***^	[2.56,3.69]
**Smoking habits** (Ref: Never)	2036	Ref		Ref		Ref		Ref	
Past smoker	1285	-0.29^*^	[-0.55,-0.04]	0.29	[-0.12,0.69]	-4.30	[-8.61,0.01]	-0.13	[-0.38,0.11]
Occasional smoker	181	-0.05	[-0.60,0.51]	-0.88^*^	[-1.76,-0.01]	-7.95	[-17.26,1.35]	-0.09	[-0.62,0.44]
Daily smoker	463	0.21	[-0.17,0.59]	-0.57	[-1.17,0.03]	-5.05	[-11.42,1.32]	0.21	[-0.16,0.57]

Multivariate linear regressions of upright event metrics. Presented as the unstandardised regression coefficient (B) and 95% confidence intervals [95% CI]. Mutually adjusted for all socioeconomic, lifestyle and health factors, and daily wear time. Additionally adjusted for daily step count

*N* Sub-group sample size

^*^p < 0.05, ^**^p < 0.01, ^***^p < 0.001

There was very little difference in upright events and total steps per day according to educational attainment. However, participants with the highest qualification recorded fewer stepping events per day (-8.07 [-12.56,-3.59] n), but each event was longer (1.42 [0.75,2.10] s) and contained more steps (2.13 [0.83,3.43] steps) than people without educational qualifications (Table 3). The main difference between people with different levels of disability was in the total number of steps taken per day. The most disabled people took an average of 1271 steps less per day than more abled people (Table 3). Similarly, there were only weak associations between characteristics of upright events and self-rated health. By contrast, the worse a person’s self-rated health, the fewer total steps they recorded each day; they recorded more stepping events overall, but they tended to be shorter and at a lower cadence, compared to people reporting better health (Table 3). In other words, people in poorer health undertook fewer sustained periods of stepping.

People with a higher BMI stood up less often than people with a healthy BMI (overweight -419.58 [-675.23,-163.93] steps; obese -1232.63 [-1518.66,-946.59] steps), their upright events were longer in duration on average, had more steps, and were less bursty (overweight -0.02 [-0.02,-0.01] *B*_n_; obese -0.03 [-0.04,-0.02] *B*_n_) (Tables 2 and 4). When they were stood up, they had a higher stepping proportion (overweight 0.62 [0.17,1.07] %; obese 0.81 [0.31,1.32] %) (Table 4). However, a higher BMI was associated with considerably fewer total steps per day, accumulated at a lower cadence for those who were obese (-0.69 [-1.34,-0.03] steps·min^−1^).

There were no differences in the frequency of upright events by occupational activity, but people in more active occupations were upright for longer each day, because the duration of each of their upright events was longer compared with sedentary occupations (Tables 2 and 4). Their pattern of being upright was more bursty than sedentary workers (standing 0.04 [0.03,0.05] *B*_n_; physical work 0.05 [0.04,0.05] *B*_n_; heavy manual 0.06 [0.04,0.07] *B*_n_), as was their patten of sedentary events (standing 0.02 [0.01,0.02] *B*_n_; physical work 0.02 [0.01,0.03] *B*_n_; heavy manual 0.03 [0.01,0.04] *B*_n_). Active workers recorded more stepping events per day and per upright events leading to a higher daily step count. Although each upright event contained more stepping events than sedentary workers, the events were longer and step rate was lower compared to the stepping rate of sedentary workers.

Daily smokers were upright more than non-smokers, and a greater proportion of upright time was standing compared to stepping, resulting in a lower daily step count.

The individual upright and stepping event durations, step count, step events, stepping proportion, and step-weighted cadence distributions are shown in histograms (Additional file 1).

### Sensitivity analyses

Upon conducting sensitivity analyses by excluding participants with an EU-SILC disability classification of ‘severely hampered’ (*n* = 103) and subsequently excluding both ‘some extent’ and ‘severely hampered’ (*n* = 491), it was observed that the overall interpretation of the results remained largely consistent. Although certain values within a categorical variable changed, the fundamental conclusions drawn from the analyses remained unaffected (Additional file 2).

## Discussion

This study aimed to describe accumulation patterns of upright and stepping events in middle-aged adults according to sociodemographic and health related factors. On average participants stood up 52.9 ± 15.3 times a day and were upright for an average 6.4 ± 1.9 h·d^−1^, similar to previous studies that reported free-living sit-to-stand transitions. The majority of upright events comprised more standing than stepping (35.6 ± 6.1% stepping) and were characterised by intermittent standing and stepping rather than continuous standing or stepping. Upright events were not uniformly distributed across the day but tended to occur in bursts. The duration of the events also varied with the typical event duration lasting just 8.0 ± 3.7 min. Overall, participants accumulated 9389 ± 3586 steps·d^−1^ with an average 198 ± 70 stepping events per day, an average of 44.1 ± 16.2 steps per event, and a step-weight average cadence 89.5 ± 8.8 steps·min^−1^. Previous studies employing thigh worn accelerometers in middle-aged populations have reported similar frequencies of upright events (either as sit-to-stand transitions, sedentary breaks, or sitting interruptions) [18, 27, 28]; whereas devices located at the hip or waist have tended to report higher frequencies [29–31]. Though, wrist worn devices have recently demonstrated good agreement with the activPAL algorithm [32]. Average duration of upright events were similar to those that have previously been reported [18, 29].

People with the same number and total time spent in upright postures can vary considerably in the composition of standing and stepping. Likewise, people recording the same total daily step count can accumulate the steps in many different ways; differences which are likely to moderate the relationship between total daily steps and health outcomes. In agreement with Blankenship et al. [18], upright events cannot all be treated the same for the purposes of studying the relationship between interruptions in sitting postures and health outcomes. However, in addition to Blankenship, this study also shows that it is insufficient to only report the average duration of upright events, the duration of stepping time within the event, and the average time between events, because the temporal distribution of the upright events and how sustained or intermittent stepping is can also vary when people have the same average duration of upright events, the same mean duration of stepping time and the same average time between events. Furthermore, this study showed that these associations persist even when adjusting for total daily step count.

This study highlights that the time spent upright is made up of varying combinations of stepping and standing and that the time spent stepping, within an upright event, can be comprised of a single sustained stepping event or multiple short stepping events interspersed with periods of standing. This also means that the same average cadence of the steps within an upright event could be based on a single stepping event done at the same step rate or multiple stepping events each with its own cadence ranging from high to low. Current cadence-based metrics typical report time or number of steps above set step-rates [33]; and associations with health outcomes do not always remain after adjusting for total volume [34, 35]. Weighting cadence by steps per stepping event, is a simple way of accounting for all steps when examining associations between step-rate and health outcomes.

The burstiness of upright events (how clustered vs evenly disbursed across the day they are) [7] in this study revealed that events are often clustered together followed by longer periods of sedentary time. In addition, sedentary event burstiness (the variation in the duration of the upright events) suggests that some people have more uniform upright durations, while others have more variation. It is highly unlikely that people will only have long sustained upright events, so the most uniform patterns of duration are likely to reflect people who are only upright for short periods – a more transient pattern of being upright. The fragmentation (measured by others by the active-to-sedentary transition probability) of upright events has been shown to be associated with health outcomes regardless of volume of activity [8, 17]. Therefore, these new metrics which characterise the number and temporal distribution of events, in addition to the variance of event durations, and the composition of standing and stepping, provide new knowledge about how people accumulate daily values of standing and stepping through different patterns.

This study highlights that key demographic and health factors are characterised by distinct postural and stepping phenotypes that may be differentially associated with health outcomes. These differences in the pattern of upright events and accumulation of steps would be expected to moderate any observed relationships between total daily steps and health outcomes [8, 14–16, 34]. For example, patterns of posture and stepping varied considerably by occupational physical activity. More active occupations were characterised by more time being upright, accumulated in both higher upright and sedentary event bursts compared to sedentary occupations. The upright time was composed of a higher proportion of standing than sedentary occupations, but a greater number of shorter and slower stepping events. This type of work pattern may partly explain why studies comparing the association of occupational activity and leisure time activity on health outcomes, find that for the same volume of activity, occupational activity is less healthy [36]. If people in sedentary occupations get more of their activity from less frequent, but longer, more intense and sustained periods of physical activity during their non-work time, then they would be expected to have better health outcomes even if they have the same volume of activity. This supports the suggestion of others that occupational activity may be insufficiently intense [36], but also highlights that observed differences may be due to different patterns of accumulation.

Patterns of activity that are characterised by frequent transient/fragmented durations have consistently been associated with a range of health outcomes including fatigue, heart failure, physical function, cognitive impairment, and mortality, independent of the total volume of physical activity [15–17, 37, 38]. This study adds to these findings by describing a new dimension to the way in which postural activity is accumulated – the burstiness of upright events [7, 39, 40]. High bursty scores indicate a daily pattern of upright events clustered together in short bursts followed by long periods of being sedentary. Whilst the burstiness metric has not been studied in aetiological studies of physical activity and health, a phenotype of both bursty and fragmented upright postures accompanied by intermittent, rather than sustained periods of stepping is likely to be associated with a loss of capacity and less confidence about undertaking sustained periods of activity.

The findings of this study have important implications for research, as much of the variation in the accumulation of activity events described here, is masked by analytical approaches which aggregate posture, stepping and standing over time or simply count/sum upright events [11, 18]. The novel phenotypes identified will help to advance research into physical activity and health, and healthy ageing, and could inform future intervention designs and policy decisions. The findings highlight why simple aggregate measures of posture and stepping can mask important variations in behaviour and why future studies cannot afford to ignore patterns of accumulation.

This study is not without limitations. Accelerometers are not direct measures of behaviour but rather a proxy. Many, including the activPAL, rely on proprietary algorithms to translate the accelerometer signal into behavioural information, which is then further processed to derive outcome metrics of interest [41, 42]. In addition, algorithm versions may change over time; it is important to note we used the activPAL CREA algorithm, which may not be comparable with the VANE algorithm, particularly with regards to transitions between sedentary and upright postures [43]. Detection of valid wake and sleep times using accelerometers is challenging, with disagreement between currently available algorithms [44]. We employed a simple and pragmatic method to identify and characterise waking wear time but, like other wake/sleep time algorithms, it is challenging to assess criterion validity against a true gold-standard, and as such there may have been some misclassification [44, 45]. The particular accelerometer used in this study may underestimate step count at slower paced walking steps, potentially leading to an overestimate of stepping cadence [46]. The resolution used to categorise postures (activPAL software recommended minimum 10 s) may also be shorter than they actually take place, leading to misclassification of the time spent in different postures. Whilst not necessarily a limitation, the age of the sample (all participants were 46 years old) has likely led to an underestimate of the true level of variation in the measures reported in this study. A wider age range, that included older people, might be expected to show greater variation. BCS70 is a rich dataset, and access to the raw accelerometer files allowed us to look beyond the aggregate measures of standing and stepping from previous studies using BCS70 summary data [47, 48]. was a strength of this study. However, the cross-sectional design of this study means we cannot determine causality. As previously described, participants who declined to wear an accelerometer were more likely to be male, smokers, report poorer health, and have a higher BMI, limiting the generalisability of our findings [49]. Finally, a recent systematic review of associations between accelerometer-measured physical activity patterns and health outcome noted the lack of adjustment for total volume of physical activity in most studies [19]; this study demonstrated associations persisted after adjustment for daily step count.

## Conclusions

This study has revealed novel phenotypes of standing and sitting that go beyond simply describing average amounts and durations. Population sub-groups display different compositions and patterns of upright events that may have important relations with functional and health outcomes. The findings start to provide explanations for why particular population sub-groups appear to have different health outcomes but seemingly record the same volume of physical activity. The study lays the groundwork for future studies to investigate how different sitting and standing phenotypes can add to our understanding of the relationship between physical activity and health. Moreover, this research could also encourage future research on physical activity interventions to explore more outcome measures than just between group differences in time spent at pre-determined intensities.

### Supplementary Information

**Additional file 1: Fig. S1.** Histograms of the composition metrics of all 1.64 million upright events; (A) upright duration (mins); (B) Stepping event duration (mins): (C) Step count per upright event (n); (D) Step events per upright event (n); (E) Step-weighted mean cadence per upright event (steps/min); (F) Upright event stepping proportion (%).

**Additional file 2: Table S1.** Sensitivity analyses regressions (excluding EU-SILC severely hampered). **Table S2.** Sensitivity analyses regressions (excluding EU-SILC severely hampered). **Table S3.** Sensitivity analyses regressions (excluding EU-SILC severely hampered and some extent). **Table S4.** Sensitivity analyses regressions (excluding EU-SILC severely hampered and some extent).

## Data Availability

The datasets analysed during the current study are available on request from the University College London, UCL Institute of Education, Centre for Longitudinal Studies (2023). 1970 British Cohort Study [data series] 10th Release. UK Data Service SN: 200001, 10.5255/UKDA-Series-200001. The Stata syntax used to derive the metrics in this study is available via GitHub repository: https://github.com/josh-culverhouse/Upright-Events.

## Abbreviations

BMI: Body mass index
BCS70: 1970 British Cohort Study
NS-SEC: National Statistics Socio-economic Classification
EU-SILC: European Union Statistics on Income and Living Conditions
