# Supplementary material for: Unravelling upright events: a descriptive epidemiology of the behavioural composition and temporal distribution of upright events in participants from the 1970 British Cohort Study
Source: BMC Public Health. 2024 Feb 21;24:535. doi: 10.1186/s12889-024-17976-2 (PMC10880236; doi:10.1186/s12889-024-17976-2)
Supplement: Supplementary file 1 — Additional file 1: Fig. S1. Histograms of the composition metrics of all 1.64 million upright events; (A) upright duration (mins); (B) Stepping event duration (mins): (C) Step count per upright event (n); (D) Step events per upright event (n); (E) Step-weighted mean cadence per upright event (steps/min); (F) Upright event stepping proportion (%). [file 12889_2024_17976_MOESM1_ESM.docx]

**
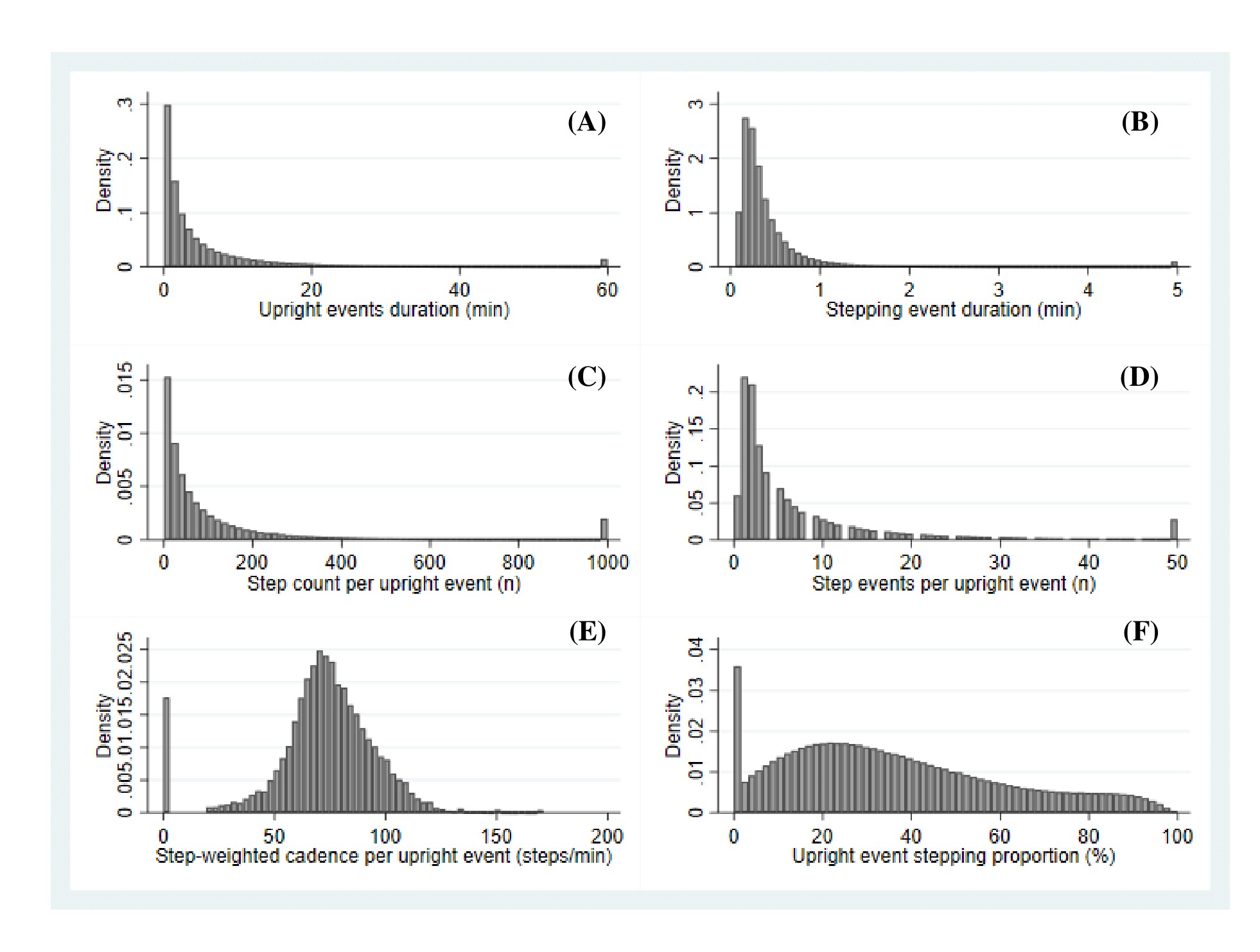
**

**Fig. s1.** Histograms of the composition metrics of all 1.64 million upright events; (A) upright duration (mins); (B) Stepping event duration (mins): (C) Step count per upright event (n); (D) Step events per upright event (n); (E) Step-weighted mean cadence per upright event (steps/min); (F) Upright event stepping proportion (%).
