# Supplementary material for: Unravelling upright events: a descriptive epidemiology of the behavioural composition and temporal distribution of upright events in participants from the 1970 British Cohort Study
Source: BMC Public Health. 2024 Feb 21;24:535. doi: 10.1186/s12889-024-17976-2 (PMC10880236; doi:10.1186/s12889-024-17976-2)
Supplement: Supplementary file 2 — Additional file 2: Table S1. Sensitivity analyses regressions (excluding EU-SILC severely hampered). Table S2. Sensitivity analyses regressions (excluding EU-SILC severely hampered). Table S3. Sensitivity analyses regressions (excluding EU-SILC severely hampered and some extent). Table S4. Sensitivity analyses regressions (excluding EU-SILC severely hampered and some extent). [file 12889_2024_17976_MOESM2_ESM.docx]

| Table. s1. Sensitivity analyses regressions (excluding EU-SILC severely hampered). | | | | | | | | | | | | | | | | |
| --- | --- | --- | --- | --- | --- | --- | --- | --- | --- | --- | --- | --- | --- | --- | --- | --- |
|  | (1) |  | (2) |  | (3) |  | (4) |  | (5) |  | (6) |  | (7) |  | (8) |  |
|  | N  upev |  | Upr  dur_h |  | std_  dur_h |  | Stp  dur_h |  | N  stpev |  | Dur  stpev |  | stps_per  stpev |  | Stpw  cad |  |
| 0.Sex | 0.00 | [0.00,0.00] | 0.00 | [0.00,0.00] | 0.00 | [0.00,0.00] | 0.00 | [0.00,0.00] | 0.00 | [0.00,0.00] | 0.00 | [0.00,0.00] | 0.00 | [0.00,0.00] | 0.00 | [0.00,0.00] |
| 1.Sex | 4.41^***^ | [3.43,5.38] | 0.37^***^ | [0.28,0.46] | 0.37^***^ | [0.29,0.46] | -0.00 | [-0.02,0.01] | 12.75^***^ | [9.64,15.86] | -3.05^***^ | [-3.51,-2.59] | -4.24^***^ | [-5.13,-3.35] | 1.04^***^ | [0.54,1.54] |
| 0.Qual | 0.00 | [0.00,0.00] | 0.00 | [0.00,0.00] | 0.00 | [0.00,0.00] | 0.00 | [0.00,0.00] | 0.00 | [0.00,0.00] | 0.00 | [0.00,0.00] | 0.00 | [0.00,0.00] | 0.00 | [0.00,0.00] |
| 1.Qual | 0.00 | [-1.24,1.25] | -0.01 | [-0.13,0.11] | -0.01 | [-0.13,0.10] | 0.00 | [-0.01,0.02] | 0.05 | [-3.90,4.00] | 0.11 | [-0.48,0.69] | 0.08 | [-1.06,1.21] | 0.13 | [-0.51,0.77] |
| 2.Qual | -0.02 | [-1.52,1.47] | -0.01 | [-0.15,0.13] | -0.01 | [-0.15,0.12] | 0.01 | [-0.01,0.02] | -1.80 | [-6.56,2.95] | 0.41 | [-0.30,1.11] | 0.40 | [-0.97,1.77] | -0.09 | [-0.86,0.68] |
| 3.Qual | -0.78 | [-2.16,0.60] | -0.13 | [-0.26,0.00] | -0.12 | [-0.24,0.00] | -0.01 | [-0.03,0.01] | -7.98^***^ | [-12.36,-3.59] | 1.41^***^ | [0.76,2.07] | 2.12^***^ | [0.86,3.38] | 0.41 | [-0.30,1.12] |
| 0.Disab | 0.00 | [0.00,0.00] | 0.00 | [0.00,0.00] | 0.00 | [0.00,0.00] | 0.00 | [0.00,0.00] | 0.00 | [0.00,0.00] | 0.00 | [0.00,0.00] | 0.00 | [0.00,0.00] | 0.00 | [0.00,0.00] |
| 1.Disab | -0.29 | [-1.88,1.30] | 0.06 | [-0.09,0.21] | 0.06 | [-0.09,0.20] | 0.00 | [-0.02,0.02] | -2.13 | [-7.17,2.91] | 0.21 | [-0.53,0.96] | 0.27 | [-1.18,1.72] | -0.18 | [-0.99,0.64] |
| 0.SRhealth | 0.00 | [0.00,0.00] | 0.00 | [0.00,0.00] | 0.00 | [0.00,0.00] | 0.00 | [0.00,0.00] | 0.00 | [0.00,0.00] | 0.00 | [0.00,0.00] | 0.00 | [0.00,0.00] | 0.00 | [0.00,0.00] |
| 1.SRhealth | 0.77 | [-0.45,1.99] | 0.16^**^ | [0.04,0.27] | 0.12^*^ | [0.01,0.23] | 0.03^***^ | [0.02,0.05] | 9.56^***^ | [5.67,13.44] | -1.44^***^ | [-2.02,-0.87] | -2.90^***^ | [-4.02,-1.79] | -1.38^***^ | [-2.01,-0.75] |
| 2.SRhealth | 0.79 | [-0.58,2.15] | 0.13^*^ | [0.00,0.26] | 0.10 | [-0.02,0.23] | 0.03^***^ | [0.01,0.05] | 10.60^***^ | [6.26,14.94] | -1.61^***^ | [-2.26,-0.97] | -3.23^***^ | [-4.47,-1.98] | -1.61^***^ | [-2.31,-0.91] |
| 3.SRhealth | 0.37 | [-1.51,2.25] | 0.18^*^ | [0.01,0.36] | 0.14 | [-0.03,0.31] | 0.05^***^ | [0.02,0.07] | 13.20^***^ | [7.22,19.17] | -1.66^***^ | [-2.55,-0.77] | -3.48^***^ | [-5.20,-1.76] | -2.32^***^ | [-3.29,-1.36] |
| 4.SRhealth | -1.47 | [-5.96,3.01] | -0.15 | [-0.57,0.28] | -0.15 | [-0.55,0.25] | 0.00 | [-0.06,0.06] | 2.33 | [-11.93,16.58] | 0.03 | [-2.08,2.15] | -0.16 | [-4.26,3.94] | -1.30 | [-3.60,1.01] |
| 0.NSSEC_3 | 0.00 | [0.00,0.00] | 0.00 | [0.00,0.00] | 0.00 | [0.00,0.00] | 0.00 | [0.00,0.00] | 0.00 | [0.00,0.00] | 0.00 | [0.00,0.00] | 0.00 | [0.00,0.00] | 0.00 | [0.00,0.00] |
| 1.NSSEC_3 | 1.96^***^ | [0.82,3.10] | 0.23^***^ | [0.12,0.33] | 0.19^***^ | [0.09,0.29] | 0.04^***^ | [0.03,0.05] | 6.99^***^ | [3.37,10.62] | -0.99^***^ | [-1.53,-0.46] | -2.18^***^ | [-3.22,-1.14] | -1.26^***^ | [-1.84,-0.67] |
| 2.NSSEC_3 | -0.35 | [-1.92,1.22] | 0.11 | [-0.04,0.26] | 0.07 | [-0.07,0.21] | 0.04^***^ | [0.02,0.06] | 3.78 | [-1.22,8.78] | -0.37 | [-1.12,0.37] | -1.21 | [-2.64,0.23] | -1.19^**^ | [-2.00,-0.39] |
| 3.NSSEC_3 | -6.24 | [-17.62,5.14] | -1.14^*^ | [-2.22,-0.07] | -1.03^*^ | [-2.04,-0.02] | -0.11 | [-0.26,0.03] | -22.90 | [-59.07,13.27] | 2.51 | [-2.86,7.88] | 5.25 | [-5.15,15.64] | 3.35 | [-2.50,9.19] |
| 4.NSSEC_3 | 0.72 | [-1.39,2.83] | 0.26^*^ | [0.06,0.46] | 0.22^*^ | [0.03,0.41] | 0.04^**^ | [0.02,0.07] | 7.86^*^ | [1.16,14.57] | -1.40^**^ | [-2.39,-0.40] | -3.04^**^ | [-4.97,-1.12] | -1.74^**^ | [-2.83,-0.66] |
| 0.BMIC | 0.00 | [0.00,0.00] | 0.00 | [0.00,0.00] | 0.00 | [0.00,0.00] | 0.00 | [0.00,0.00] | 0.00 | [0.00,0.00] | 0.00 | [0.00,0.00] | 0.00 | [0.00,0.00] | 0.00 | [0.00,0.00] |
| 1.BMIC | -3.15^***^ | [-4.26,-2.05] | -0.10 | [-0.21,0.00] | -0.10 | [-0.19,0.00] | -0.01 | [-0.02,0.01] | -0.96 | [-4.49,2.56] | 0.20 | [-0.32,0.73] | 0.29 | [-0.72,1.30] | -0.14 | [-0.71,0.43] |
| 2.BMIC | -6.48^***^ | [-7.73,-5.22] | -0.12^*^ | [-0.24,-0.00] | -0.11 | [-0.22,0.01] | -0.01 | [-0.03,0.00] | -2.67 | [-6.66,1.31] | 0.47 | [-0.12,1.06] | 0.62 | [-0.52,1.77] | -0.58 | [-1.22,0.07] |
| 3.BMIC | -13.10^***^ | [-16.08,-10.13] | -0.03 | [-0.31,0.25] | -0.00 | [-0.27,0.26] | -0.02 | [-0.06,0.02] | -6.85 | [-16.31,2.61] | 1.19 | [-0.22,2.59] | 1.43 | [-1.29,4.15] | -1.61^*^ | [-3.14,-0.08] |
| 4.BMIC | -8.26^***^ | [-11.58,-4.95] | -0.08 | [-0.39,0.24] | -0.07 | [-0.37,0.22] | -0.01 | [-0.05,0.04] | 0.83 | [-9.71,11.37] | 0.21 | [-1.36,1.77] | -0.10 | [-3.13,2.93] | -1.35 | [-3.05,0.36] |
| 0.OccAct | 0.00 | [0.00,0.00] | 0.00 | [0.00,0.00] | 0.00 | [0.00,0.00] | 0.00 | [0.00,0.00] | 0.00 | [0.00,0.00] | 0.00 | [0.00,0.00] | 0.00 | [0.00,0.00] | 0.00 | [0.00,0.00] |
| 1.OccAct | -0.60 | [-1.92,0.72] | 1.18^***^ | [1.06,1.31] | 1.08^***^ | [0.96,1.20] | 0.10^***^ | [0.09,0.12] | 29.10^***^ | [24.90,33.30] | -3.97^***^ | [-4.59,-3.35] | -7.87^***^ | [-9.08,-6.66] | -3.76^***^ | [-4.44,-3.08] |
| 2.OccAct | -0.43 | [-1.66,0.80] | 1.06^***^ | [0.94,1.17] | 0.91^***^ | [0.80,1.02] | 0.15^***^ | [0.13,0.16] | 35.17^***^ | [31.25,39.09] | -4.56^***^ | [-5.15,-3.98] | -9.65^***^ | [-10.78,-8.53] | -5.80^***^ | [-6.44,-5.17] |
| 3.OccAct | -0.06 | [-2.44,2.31] | 1.28^***^ | [1.05,1.50] | 1.03^***^ | [0.82,1.24] | 0.25^***^ | [0.22,0.28] | 51.86^***^ | [44.31,59.41] | -6.16^***^ | [-7.28,-5.04] | -13.51^***^ | [-15.68,-11.34] | -8.58^***^ | [-9.80,-7.36] |
| 0.Smoking | 0.00 | [0.00,0.00] | 0.00 | [0.00,0.00] | 0.00 | [0.00,0.00] | 0.00 | [0.00,0.00] | 0.00 | [0.00,0.00] | 0.00 | [0.00,0.00] | 0.00 | [0.00,0.00] | 0.00 | [0.00,0.00] |
| 1.Smoking | 1.51^**^ | [0.50,2.52] | -0.11^*^ | [-0.20,-0.01] | -0.12^*^ | [-0.21,-0.03] | 0.01 | [-0.00,0.02] | 0.14 | [-3.07,3.35] | -0.04 | [-0.51,0.44] | -0.23 | [-1.15,0.69] | -0.30 | [-0.82,0.22] |
| 2.Smoking | 2.53^*^ | [0.35,4.72] | 0.24^*^ | [0.03,0.45] | 0.24^*^ | [0.04,0.43] | 0.00 | [-0.03,0.03] | 3.88 | [-3.07,10.83] | -0.70 | [-1.73,0.33] | -0.96 | [-2.95,1.04] | 0.01 | [-1.11,1.14] |
| 3.Smoking | 2.90^***^ | [1.38,4.41] | 0.20^**^ | [0.06,0.34] | 0.18^*^ | [0.04,0.31] | 0.02^*^ | [0.00,0.04] | 9.82^***^ | [5.00,14.64] | -1.22^***^ | [-1.94,-0.51] | -2.20^**^ | [-3.59,-0.82] | -1.26^**^ | [-2.04,-0.48] |
| wake_time_h | 2.25^***^ | [1.74,2.76] | 0.24^***^ | [0.19,0.29] | 0.22^***^ | [0.18,0.27] | 0.02^***^ | [0.01,0.03] | 7.95^***^ | [6.33,9.57] | -0.93^***^ | [-1.17,-0.69] | -1.69^***^ | [-2.15,-1.22] | -0.65^***^ | [-0.91,-0.39] |
| daily_n_stps | 0.00^***^ | [0.00,0.00] | 0.00^***^ | [0.00,0.00] | 0.00^***^ | [0.00,0.00] | 0.00^***^ | [0.00,0.00] | 0.01^***^ | [0.01,0.01] | 0.00^***^ | [0.00,0.00] | 0.00^***^ | [0.00,0.00] | 0.00^***^ | [0.00,0.00] |
| _cons | 11.10^**^ | [2.76,19.45] | -0.74 | [-1.52,0.05] | -0.59 | [-1.33,0.15] | -0.15^**^ | [-0.25,-0.04] | -73.25^***^ | [-99.76,-46.73] | 38.35^***^ | [34.41,42.29] | 57.64^***^ | [50.02,65.26] | 95.15^***^ | [90.86,99.43] |
| p | 0.00 |  | 0.00 |  | 0.00 |  | 0.00 |  | 0.00 |  | 0.00 |  | 0.00 |  | 0.00 |  |
| 95% confidence intervals in brackets  ^*^ *p* < 0.05, ^**^ *p* < 0.01, ^***^ *p* < 0.001 | | | | | | | | | | | | | | | | |

Additional file 2

| Table. s2. Sensitivity analyses regressions (excluding EU-SILC severely hampered). | | | | | | | | | | | | | |
| --- | --- | --- | --- | --- | --- | --- | --- | --- | --- | --- | --- | --- | --- |
|  | (1) |  | (2) |  | (3) |  | (4) |  | (8) |  | (9) |  |  |
|  | prop_stp  _to_std |  | Upev  dur_min |  | Upev  n_stpev |  | Upev  n_stps |  | Upev  bursti |  | Nonupev  bursti |  |  |
| 0.Sex | 0.00 | [0.00,0.00] | 0.00 | [0.00,0.00] | 0.00 | [0.00,0.00] | 0.00 | [0.00,0.00] | 0.00 | [0.00,0.00] | 0.00 | [0.00,0.00] |  |
| 1.Sex | -0.12 | [-0.51,0.26] | -0.26^*^ | [-0.51,-0.02] | -0.06 | [-0.30,0.17] | -17.84^***^ | [-21.95,-13.73] | 0.05^***^ | [0.04,0.05] | -0.00 | [-0.01,0.00] |  |
| 0.Qual | 0.00 | [0.00,0.00] | 0.00 | [0.00,0.00] | 0.00 | [0.00,0.00] | 0.00 | [0.00,0.00] | 0.00 | [0.00,0.00] | 0.00 | [0.00,0.00] |  |
| 1.Qual | 0.02 | [-0.47,0.51] | -0.08 | [-0.39,0.23] | -0.03 | [-0.33,0.27] | -2.03 | [-7.26,3.19] | 0.00 | [-0.00,0.01] | -0.01 | [-0.01,0.00] |  |
| 2.Qual | -0.36 | [-0.95,0.23] | -0.14 | [-0.52,0.23] | -0.19 | [-0.55,0.17] | -2.49 | [-8.78,3.80] | 0.00 | [-0.00,0.01] | -0.01^*^ | [-0.02,-0.00] |  |
| 3.Qual | 0.00 | [-0.54,0.55] | -0.17 | [-0.52,0.17] | -0.32 | [-0.65,0.01] | 0.02 | [-5.78,5.81] | -0.00 | [-0.01,0.01] | -0.02^***^ | [-0.03,-0.01] |  |
| 0.Disab | 0.00 | [0.00,0.00] | 0.00 | [0.00,0.00] | 0.00 | [0.00,0.00] | 0.00 | [0.00,0.00] | 0.00 | [0.00,0.00] | 0.00 | [0.00,0.00] |  |
| 1.Disab | -0.65^*^ | [-1.28,-0.03] | 0.31 | [-0.09,0.71] | 0.21 | [-0.17,0.59] | 3.30 | [-3.36,9.97] | 0.00 | [-0.00,0.01] | -0.00 | [-0.01,0.01] |  |
| 0.SRhealth | 0.00 | [0.00,0.00] | 0.00 | [0.00,0.00] | 0.00 | [0.00,0.00] | 0.00 | [0.00,0.00] | 0.00 | [0.00,0.00] | 0.00 | [0.00,0.00] |  |
| 1.SRhealth | 0.23 | [-0.25,0.72] | 0.09 | [-0.22,0.39] | 0.32^*^ | [0.03,0.62] | -2.58 | [-7.72,2.55] | 0.01 | [-0.00,0.01] | 0.00 | [-0.00,0.01] |  |
| 2.SRhealth | 0.25 | [-0.28,0.79] | 0.05 | [-0.29,0.39] | 0.30 | [-0.02,0.63] | -1.99 | [-7.74,3.75] | 0.01^*^ | [0.00,0.02] | 0.00 | [-0.01,0.01] |  |
| 3.SRhealth | -0.15 | [-0.89,0.59] | 0.45 | [-0.02,0.92] | 0.58^*^ | [0.13,1.04] | 0.27 | [-7.63,8.17] | 0.01 | [-0.00,0.02] | 0.00 | [-0.01,0.01] |  |
| 4.SRhealth | 0.82 | [-0.95,2.59] | -0.37 | [-1.49,0.76] | -0.18 | [-1.26,0.89] | 2.77 | [-16.08,21.61] | -0.02 | [-0.05,0.00] | 0.00 | [-0.02,0.03] |  |
| 0.NSSEC_3 | 0.00 | [0.00,0.00] | 0.00 | [0.00,0.00] | 0.00 | [0.00,0.00] | 0.00 | [0.00,0.00] | 0.00 | [0.00,0.00] | 0.00 | [0.00,0.00] |  |
| 1.NSSEC_3 | -0.19 | [-0.64,0.26] | 0.15 | [-0.14,0.44] | 0.37^**^ | [0.10,0.65] | -4.13 | [-8.92,0.66] | 0.01^*^ | [0.00,0.01] | 0.01 | [-0.00,0.01] |  |
| 2.NSSEC_3 | 0.07 | [-0.55,0.69] | 0.39 | [-0.01,0.78] | 0.52^**^ | [0.14,0.90] | 2.16 | [-4.45,8.77] | 0.00 | [-0.01,0.01] | 0.01^*^ | [0.00,0.02] |  |
| 3.NSSEC_3 | 1.75 | [-2.74,6.24] | -0.83 | [-3.69,2.02] | -0.68 | [-3.42,2.05] | 18.36 | [-29.47,66.18] | 0.00 | [-0.06,0.07] | -0.03 | [-0.09,0.04] |  |
| 4.NSSEC_3 | -0.40 | [-1.24,0.43] | 0.39 | [-0.14,0.91] | 0.59^*^ | [0.08,1.10] | 0.87 | [-7.99,9.74] | 0.01^*^ | [0.00,0.02] | -0.00 | [-0.01,0.01] |  |
| 0.BMIC | 0.00 | [0.00,0.00] | 0.00 | [0.00,0.00] | 0.00 | [0.00,0.00] | 0.00 | [0.00,0.00] | 0.00 | [0.00,0.00] | 0.00 | [0.00,0.00] |  |
| 1.BMIC | 0.55^*^ | [0.12,0.99] | 0.31^*^ | [0.03,0.58] | 0.26 | [-0.01,0.52] | 9.92^***^ | [5.26,14.58] | -0.02^***^ | [-0.02,-0.01] | -0.00 | [-0.01,0.01] |  |
| 2.BMIC | 0.87^***^ | [0.38,1.37] | 0.97^***^ | [0.65,1.28] | 0.84^***^ | [0.54,1.14] | 25.81^***^ | [20.55,31.08] | -0.03^***^ | [-0.04,-0.02] | -0.00 | [-0.01,0.01] |  |
| 3.BMIC | 0.92 | [-0.25,2.10] | 2.41^***^ | [1.67,3.16] | 1.90^***^ | [1.18,2.61] | 49.23^***^ | [36.72,61.73] | -0.04^***^ | [-0.06,-0.03] | 0.01 | [-0.01,0.03] |  |
| 4.BMIC | 0.49 | [-0.82,1.80] | 1.44^***^ | [0.60,2.27] | 1.37^***^ | [0.57,2.16] | 30.08^***^ | [16.15,44.02] | -0.00 | [-0.02,0.01] | 0.00 | [-0.01,0.02] |  |
| 0.OccAct | 0.00 | [0.00,0.00] | 0.00 | [0.00,0.00] | 0.00 | [0.00,0.00] | 0.00 | [0.00,0.00] | 0.00 | [0.00,0.00] | 0.00 | [0.00,0.00] |  |
| 1.OccAct | -1.53^***^ | [-2.05,-1.01] | 1.79^***^ | [1.46,2.12] | 1.98^***^ | [1.67,2.30] | 2.14 | [-3.42,7.70] | 0.04^***^ | [0.03,0.04] | 0.02^***^ | [0.01,0.02] |  |
| 2.OccAct | -1.34^***^ | [-1.82,-0.85] | 1.46^***^ | [1.15,1.77] | 2.19^***^ | [1.89,2.48] | 3.42 | [-1.76,8.60] | 0.04^***^ | [0.04,0.05] | 0.02^***^ | [0.01,0.03] |  |
| 3.OccAct | -0.98^*^ | [-1.92,-0.04] | 1.80^***^ | [1.20,2.39] | 3.09^***^ | [2.52,3.66] | 6.24 | [-3.74,16.22] | 0.06^***^ | [0.05,0.07] | 0.03^***^ | [0.01,0.04] |  |
| 0.Smoking | 0.00 | [0.00,0.00] | 0.00 | [0.00,0.00] | 0.00 | [0.00,0.00] | 0.00 | [0.00,0.00] | 0.00 | [0.00,0.00] | 0.00 | [0.00,0.00] |  |
| 1.Smoking | 0.36 | [-0.04,0.76] | -0.34^**^ | [-0.60,-0.09] | -0.16 | [-0.40,0.09] | -4.67^*^ | [-8.91,-0.42] | -0.00 | [-0.01,0.00] | -0.00 | [-0.01,0.00] |  |
| 2.Smoking | -0.71 | [-1.57,0.15] | -0.10 | [-0.65,0.45] | -0.14 | [-0.66,0.39] | -7.69 | [-16.88,1.50] | -0.01 | [-0.02,0.01] | -0.00 | [-0.02,0.01] |  |
| 3.Smoking | -0.48 | [-1.08,0.12] | 0.20 | [-0.18,0.58] | 0.23 | [-0.14,0.59] | -5.19 | [-11.57,1.18] | -0.01^*^ | [-0.02,-0.00] | -0.01^*^ | [-0.02,-0.00] |  |
| wake_time_h | -0.33^**^ | [-0.53,-0.13] | -0.01 | [-0.13,0.12] | -0.02 | [-0.15,0.10] | -8.37^***^ | [-10.50,-6.23] | 0.02^***^ | [0.02,0.02] | 0.00 | [-0.00,0.01] |  |
| avg_daily_n_stps | 0.00^***^ | [0.00,0.00] | 0.00^***^ | [0.00,0.00] | 0.00^***^ | [0.00,0.00] | 0.02^***^ | [0.02,0.02] | 0.00^***^ | [0.00,0.00] | 0.00^***^ | [0.00,0.00] |  |
| _cons | 35.69^***^ | [32.40,38.99] | 4.50^***^ | [2.40,6.59] | 4.30^***^ | [2.29,6.30] | 144.73^***^ | [109.68,179.79] | -0.08^**^ | [-0.13,-0.03] | 0.19^***^ | [0.14,0.24] |  |
| p | 0.00 |  | 0.00 |  | 0.00 |  | 0.00 |  | 0.00 |  | 0.00 |  |  |
| 95% confidence intervals in brackets  ^*^ *p* < 0.05, ^**^ *p* < 0.01, ^***^ *p* < 0.001 | | | | | | | | | | | | | |

| Table. s3. Sensitivity analyses regressions (excluding EU-SILC severely hampered and some extent). | | | | | | | | | | | | | | | | |
| --- | --- | --- | --- | --- | --- | --- | --- | --- | --- | --- | --- | --- | --- | --- | --- | --- |
|  | (1) |  | (2) |  | (3) |  | (4) |  | (5) |  | (6) |  | (7) |  | (8) |  |
|  | N  upev |  | upr_  ur_h |  | Std  dur_h |  | Stp  dur_h |  | N  stpev |  | Dur  stpev |  | Stps  per_stpev |  | Stpw  cad |  |
| 0.Sex | 0.00 | [0.00,0.00] | 0.00 | [0.00,0.00] | 0.00 | [0.00,0.00] | 0.00 | [0.00,0.00] | 0.00 | [0.00,0.00] | 0.00 | [0.00,0.00] | 0.00 | [0.00,0.00] | 0.00 | [0.00,0.00] |
| 1.Sex | 4.26^***^ | [3.23,5.29] | 0.36^***^ | [0.27,0.46] | 0.37^***^ | [0.27,0.46] | -0.00 | [-0.01,0.01] | 13.72^***^ | [10.42,17.01] | -3.18^***^ | [-3.67,-2.68] | -4.48^***^ | [-5.43,-3.52] | 0.91^***^ | [0.38,1.44] |
| 0.Qual | 0.00 | [0.00,0.00] | 0.00 | [0.00,0.00] | 0.00 | [0.00,0.00] | 0.00 | [0.00,0.00] | 0.00 | [0.00,0.00] | 0.00 | [0.00,0.00] | 0.00 | [0.00,0.00] | 0.00 | [0.00,0.00] |
| 1.Qual | -0.42 | [-1.73,0.89] | 0.02 | [-0.11,0.14] | 0.01 | [-0.11,0.13] | 0.01 | [-0.01,0.02] | 0.19 | [-4.00,4.37] | 0.11 | [-0.51,0.74] | 0.04 | [-1.17,1.26] | 0.07 | [-0.60,0.75] |
| 2.Qual | -0.07 | [-1.65,1.51] | -0.01 | [-0.16,0.14] | -0.02 | [-0.16,0.12] | 0.01 | [-0.01,0.03] | -1.85 | [-6.88,3.18] | 0.41 | [-0.34,1.17] | 0.43 | [-1.03,1.89] | 0.01 | [-0.81,0.82] |
| 3.Qual | -0.85 | [-2.30,0.59] | -0.10 | [-0.23,0.04] | -0.10 | [-0.22,0.03] | -0.00 | [-0.02,0.02] | -6.78^**^ | [-11.40,-2.16] | 1.27^***^ | [0.58,1.96] | 1.75^*^ | [0.41,3.09] | 0.18 | [-0.57,0.92] |
| 0.Disab | 0.00 | [0.00,0.00] | 0.00 | [0.00,0.00] | 0.00 | [0.00,0.00] | 0.00 | [0.00,0.00] | 0.00 | [0.00,0.00] | 0.00 | [0.00,0.00] | 0.00 | [0.00,0.00] | 0.00 | [0.00,0.00] |
| 0.SRhealth | 0.00 | [0.00,0.00] | 0.00 | [0.00,0.00] | 0.00 | [0.00,0.00] | 0.00 | [0.00,0.00] | 0.00 | [0.00,0.00] | 0.00 | [0.00,0.00] | 0.00 | [0.00,0.00] | 0.00 | [0.00,0.00] |
| 1.SRhealth | 0.62 | [-0.62,1.86] | 0.15^*^ | [0.04,0.27] | 0.12^*^ | [0.01,0.23] | 0.03^***^ | [0.02,0.05] | 9.52^***^ | [5.57,13.48] | -1.47^***^ | [-2.07,-0.88] | -2.97^***^ | [-4.11,-1.82] | -1.40^***^ | [-2.04,-0.76] |
| 2.SRhealth | 0.93 | [-0.48,2.34] | 0.15^*^ | [0.01,0.28] | 0.11 | [-0.01,0.24] | 0.03^***^ | [0.01,0.05] | 11.17^***^ | [6.68,15.65] | -1.72^***^ | [-2.40,-1.05] | -3.40^***^ | [-4.71,-2.10] | -1.64^***^ | [-2.36,-0.91] |
| 3.SRhealth | 0.49 | [-1.61,2.58] | 0.13 | [-0.06,0.33] | 0.10 | [-0.09,0.28] | 0.04^**^ | [0.01,0.06] | 12.34^***^ | [5.66,19.02] | -1.73^***^ | [-2.73,-0.73] | -3.53^***^ | [-5.47,-1.59] | -2.23^***^ | [-3.31,-1.15] |
| 4.SRhealth | -1.02 | [-7.06,5.02] | -0.01 | [-0.58,0.55] | -0.03 | [-0.56,0.50] | 0.02 | [-0.06,0.09] | 4.56 | [-14.70,23.83] | -1.05 | [-3.93,1.84] | -2.24 | [-7.83,3.35] | -2.04 | [-5.15,1.07] |
| 0.NSSEC_3 | 0.00 | [0.00,0.00] | 0.00 | [0.00,0.00] | 0.00 | [0.00,0.00] | 0.00 | [0.00,0.00] | 0.00 | [0.00,0.00] | 0.00 | [0.00,0.00] | 0.00 | [0.00,0.00] | 0.00 | [0.00,0.00] |
| 1.NSSEC_3 | 1.90^**^ | [0.70,3.11] | 0.25^***^ | [0.14,0.36] | 0.21^***^ | [0.10,0.31] | 0.04^***^ | [0.03,0.06] | 8.10^***^ | [4.26,11.94] | -1.09^***^ | [-1.67,-0.52] | -2.42^***^ | [-3.54,-1.31] | -1.50^***^ | [-2.12,-0.88] |
| 2.NSSEC_3 | -0.34 | [-2.00,1.32] | 0.11 | [-0.04,0.27] | 0.08 | [-0.07,0.22] | 0.04^***^ | [0.02,0.06] | 3.71 | [-1.59,9.00] | -0.40 | [-1.19,0.40] | -1.30 | [-2.84,0.23] | -1.35^**^ | [-2.20,-0.49] |
| 3.NSSEC_3 | -7.30 | [-19.77,5.17] | -1.04 | [-2.21,0.13] | -0.93 | [-2.03,0.17] | -0.11 | [-0.27,0.04] | -19.03 | [-58.81,20.76] | 1.72 | [-4.24,7.68] | 4.06 | [-7.49,15.61] | 3.41 | [-3.01,9.83] |
| 4.NSSEC_3 | 0.43 | [-1.84,2.70] | 0.22^*^ | [0.01,0.43] | 0.18 | [-0.02,0.38] | 0.04^**^ | [0.01,0.07] | 7.57^*^ | [0.32,14.82] | -1.53^**^ | [-2.62,-0.45] | -3.20^**^ | [-5.31,-1.10] | -1.67^**^ | [-2.84,-0.50] |
| 0.BMIC | 0.00 | [0.00,0.00] | 0.00 | [0.00,0.00] | 0.00 | [0.00,0.00] | 0.00 | [0.00,0.00] | 0.00 | [0.00,0.00] | 0.00 | [0.00,0.00] | 0.00 | [0.00,0.00] | 0.00 | [0.00,0.00] |
| 1.BMIC | -3.18^***^ | [-4.34,-2.02] | -0.14^*^ | [-0.25,-0.03] | -0.13^*^ | [-0.23,-0.03] | -0.01 | [-0.02,0.01] | -1.36 | [-5.07,2.34] | 0.28 | [-0.28,0.83] | 0.43 | [-0.65,1.50] | -0.11 | [-0.71,0.48] |
| 2.BMIC | -6.51^***^ | [-7.83,-5.19] | -0.14^*^ | [-0.27,-0.02] | -0.13^*^ | [-0.25,-0.01] | -0.01 | [-0.03,0.01] | -3.29 | [-7.50,0.93] | 0.59 | [-0.04,1.22] | 0.77 | [-0.45,2.00] | -0.71^*^ | [-1.39,-0.03] |
| 3.BMIC | -13.76^***^ | [-17.07,-10.46] | 0.06 | [-0.25,0.37] | 0.09 | [-0.21,0.38] | -0.03 | [-0.07,0.01] | -9.20 | [-19.74,1.35] | 1.71^*^ | [0.13,3.29] | 2.38 | [-0.68,5.44] | -1.00 | [-2.70,0.70] |
| 4.BMIC | -7.91^***^ | [-11.46,-4.35] | -0.10 | [-0.43,0.23] | -0.10 | [-0.41,0.22] | -0.00 | [-0.05,0.04] | 0.84 | [-10.49,12.17] | 0.18 | [-1.51,1.88] | -0.08 | [-3.37,3.21] | -1.18 | [-3.01,0.64] |
| 0.OccAct | 0.00 | [0.00,0.00] | 0.00 | [0.00,0.00] | 0.00 | [0.00,0.00] | 0.00 | [0.00,0.00] | 0.00 | [0.00,0.00] | 0.00 | [0.00,0.00] | 0.00 | [0.00,0.00] | 0.00 | [0.00,0.00] |
| 1.OccAct | -0.26 | [-1.65,1.13] | 1.16^***^ | [1.03,1.29] | 1.06^***^ | [0.94,1.18] | 0.10^***^ | [0.08,0.12] | 28.80^***^ | [24.37,33.23] | -3.89^***^ | [-4.56,-3.23] | -7.75^***^ | [-9.04,-6.46] | -3.66^***^ | [-4.37,-2.94] |
| 2.OccAct | -0.14 | [-1.45,1.17] | 1.10^***^ | [0.98,1.23] | 0.95^***^ | [0.84,1.07] | 0.15^***^ | [0.13,0.17] | 36.35^***^ | [32.16,40.53] | -4.67^***^ | [-5.30,-4.04] | -9.92^***^ | [-11.14,-8.71] | -5.92^***^ | [-6.60,-5.24] |
| 3.OccAct | 0.22 | [-2.26,2.70] | 1.32^***^ | [1.09,1.56] | 1.07^***^ | [0.85,1.29] | 0.25^***^ | [0.22,0.28] | 53.72^***^ | [45.82,61.62] | -6.37^***^ | [-7.55,-5.18] | -13.91^***^ | [-16.20,-11.62] | -8.71^***^ | [-9.99,-7.44] |
| 0.Smoking | 0.00 | [0.00,0.00] | 0.00 | [0.00,0.00] | 0.00 | [0.00,0.00] | 0.00 | [0.00,0.00] | 0.00 | [0.00,0.00] | 0.00 | [0.00,0.00] | 0.00 | [0.00,0.00] | 0.00 | [0.00,0.00] |
| 1.Smoking | 1.46^**^ | [0.39,2.52] | -0.14^**^ | [-0.24,-0.04] | -0.14^**^ | [-0.24,-0.05] | 0.01 | [-0.01,0.02] | 0.24 | [-3.16,3.64] | -0.01 | [-0.52,0.50] | -0.16 | [-1.14,0.83] | -0.20 | [-0.75,0.35] |
| 2.Smoking | 2.38^*^ | [0.09,4.68] | 0.27^*^ | [0.06,0.49] | 0.27^**^ | [0.06,0.47] | 0.01 | [-0.02,0.03] | 4.11 | [-3.21,11.43] | -0.69 | [-1.79,0.40] | -1.03 | [-3.15,1.09] | -0.05 | [-1.23,1.13] |
| 3.Smoking | 2.84^***^ | [1.21,4.47] | 0.14 | [-0.01,0.30] | 0.13 | [-0.02,0.27] | 0.01 | [-0.01,0.03] | 8.03^**^ | [2.83,13.23] | -1.04^**^ | [-1.82,-0.26] | -1.87^*^ | [-3.38,-0.36] | -1.23^**^ | [-2.07,-0.40] |
| wake_time_h | 1.98^***^ | [1.44,2.52] | 0.24^***^ | [0.19,0.29] | 0.22^***^ | [0.17,0.27] | 0.02^***^ | [0.01,0.03] | 7.91^***^ | [6.20,9.62] | -0.92^***^ | [-1.17,-0.66] | -1.67^***^ | [-2.16,-1.17] | -0.65^***^ | [-0.93,-0.37] |
| daily_n_stps | 0.00^***^ | [0.00,0.00] | 0.00^***^ | [0.00,0.00] | 0.00^***^ | [0.00,0.00] | 0.00^***^ | [0.00,0.00] | 0.01^***^ | [0.01,0.01] | 0.00^***^ | [0.00,0.00] | 0.00^***^ | [0.00,0.00] | 0.00^***^ | [0.00,0.00] |
| _cons | 15.55^***^ | [6.75,24.35] | -0.65 | [-1.48,0.17] | -0.51 | [-1.29,0.27] | -0.14^*^ | [-0.25,-0.03] | -71.94^***^ | [-100.01,-43.86] | 38.24^***^ | [34.03,42.44] | 57.51^***^ | [49.36,65.66] | 95.41^***^ | [90.88,99.94] |
| p | 0.00 |  | 0.00 |  | 0.00 |  | 0.00 |  | 0.00 |  | 0.00 |  | 0.00 |  | 0.00 |  |
| 95% confidence intervals in brackets  ^*^ *p* < 0.05, ^**^ *p* < 0.01, ^***^ *p* < 0.001 | | | | | | | | | | | | | | | | |

| Table. s4. Sensitivity analyses regressions (excluding EU-SILC severely hampered and some extent). | | | | | | | | | | | | |
| --- | --- | --- | --- | --- | --- | --- | --- | --- | --- | --- | --- | --- |
|  | (1) |  | (2) |  | (3) |  | (4) |  | (8) |  | (9) |  |
|  | Prop  stp_to_std |  | upev_  ur_min |  | Upev  n_stpev |  | Upev  n_stps |  | Upev  bursti |  | Nonupev  bursti |  |
| 0.Sex | 0.00 | [0.00,0.00] | 0.00 | [0.00,0.00] | 0.00 | [0.00,0.00] | 0.00 | [0.00,0.00] | 0.00 | [0.00,0.00] | 0.00 | [0.00,0.00] |
| 1.Sex | -0.05 | [-0.46,0.35] | -0.27^*^ | [-0.50,-0.04] | -0.01 | [-0.25,0.24] | -17.71^***^ | [-22.01,-13.42] | 0.05^***^ | [0.04,0.05] | -0.00 | [-0.01,0.00] |
| 0.Qual | 0.00 | [0.00,0.00] | 0.00 | [0.00,0.00] | 0.00 | [0.00,0.00] | 0.00 | [0.00,0.00] | 0.00 | [0.00,0.00] | 0.00 | [0.00,0.00] |
| 1.Qual | -0.11 | [-0.62,0.41] | 0.10 | [-0.19,0.40] | 0.09 | [-0.22,0.40] | 0.02 | [-5.43,5.48] | 0.00 | [-0.01,0.01] | -0.01 | [-0.01,0.00] |
| 2.Qual | -0.29 | [-0.91,0.33] | -0.06 | [-0.41,0.30] | -0.15 | [-0.52,0.22] | -1.46 | [-8.02,5.10] | 0.00 | [-0.01,0.01] | -0.01^*^ | [-0.02,-0.00] |
| 3.Qual | -0.01 | [-0.58,0.55] | -0.07 | [-0.39,0.26] | -0.22 | [-0.56,0.12] | 1.20 | [-4.82,7.22] | -0.00 | [-0.01,0.01] | -0.02^***^ | [-0.03,-0.01] |
| 0.Disab | 0.00 | [0.00,0.00] | 0.00 | [0.00,0.00] | 0.00 | [0.00,0.00] | 0.00 | [0.00,0.00] | 0.00 | [0.00,0.00] | 0.00 | [0.00,0.00] |
| 0.SRhealth | 0.00 | [0.00,0.00] | 0.00 | [0.00,0.00] | 0.00 | [0.00,0.00] | 0.00 | [0.00,0.00] | 0.00 | [0.00,0.00] | 0.00 | [0.00,0.00] |
| 1.SRhealth | 0.26 | [-0.23,0.74] | 0.12 | [-0.16,0.40] | 0.36^*^ | [0.07,0.65] | -2.04 | [-7.19,3.11] | 0.01 | [-0.00,0.01] | 0.00 | [-0.00,0.01] |
| 2.SRhealth | 0.26 | [-0.29,0.81] | 0.12 | [-0.19,0.44] | 0.36^*^ | [0.03,0.69] | -1.57 | [-7.42,4.29] | 0.01^*^ | [0.00,0.02] | 0.00 | [-0.01,0.01] |
| 3.SRhealth | -0.11 | [-0.93,0.71] | 0.17 | [-0.30,0.64] | 0.42 | [-0.08,0.91] | -2.33 | [-11.04,6.39] | 0.00 | [-0.01,0.02] | -0.01 | [-0.02,0.01] |
| 4.SRhealth | 1.71 | [-0.66,4.08] | 0.04 | [-1.32,1.40] | 0.10 | [-1.32,1.52] | 1.73 | [-23.39,26.85] | -0.00 | [-0.04,0.03] | -0.02 | [-0.06,0.01] |
| 0.NSSEC_3 | 0.00 | [0.00,0.00] | 0.00 | [0.00,0.00] | 0.00 | [0.00,0.00] | 0.00 | [0.00,0.00] | 0.00 | [0.00,0.00] | 0.00 | [0.00,0.00] |
| 1.NSSEC_3 | -0.23 | [-0.70,0.25] | 0.18 | [-0.09,0.45] | 0.42^**^ | [0.14,0.70] | -3.85 | [-8.85,1.15] | 0.01^*^ | [0.00,0.02] | 0.00 | [-0.00,0.01] |
| 2.NSSEC_3 | 0.08 | [-0.57,0.73] | 0.19 | [-0.18,0.57] | 0.40^*^ | [0.01,0.79] | 0.59 | [-6.31,7.49] | 0.00 | [-0.01,0.01] | 0.01 | [-0.00,0.02] |
| 3.NSSEC_3 | 1.41 | [-3.49,6.31] | -0.54 | [-3.34,2.27] | -0.37 | [-3.30,2.56] | 23.95 | [-27.91,75.81] | -0.03 | [-0.10,0.05] | -0.04 | [-0.12,0.03] |
| 4.NSSEC_3 | -0.32 | [-1.21,0.57] | 0.36 | [-0.15,0.87] | 0.58^*^ | [0.04,1.11] | 1.45 | [-8.00,10.91] | 0.01 | [-0.00,0.03] | -0.01 | [-0.02,0.01] |
| 0.BMIC | 0.00 | [0.00,0.00] | 0.00 | [0.00,0.00] | 0.00 | [0.00,0.00] | 0.00 | [0.00,0.00] | 0.00 | [0.00,0.00] | 0.00 | [0.00,0.00] |
| 1.BMIC | 0.60^**^ | [0.15,1.06] | 0.23 | [-0.03,0.49] | 0.20 | [-0.07,0.48] | 9.86^***^ | [5.04,14.69] | -0.02^***^ | [-0.02,-0.01] | 0.00 | [-0.01,0.01] |
| 2.BMIC | 0.88^***^ | [0.36,1.40] | 0.86^***^ | [0.57,1.16] | 0.78^***^ | [0.47,1.10] | 25.52^***^ | [20.02,31.01] | -0.03^***^ | [-0.04,-0.02] | 0.00 | [-0.01,0.01] |
| 3.BMIC | 0.22 | [-1.08,1.52] | 2.77^***^ | [2.03,3.51] | 2.04^***^ | [1.26,2.82] | 53.22^***^ | [39.47,66.96] | -0.04^***^ | [-0.06,-0.02] | 0.01 | [-0.00,0.03] |
| 4.BMIC | 0.45 | [-0.95,1.85] | 1.33^**^ | [0.53,2.13] | 1.41^***^ | [0.57,2.24] | 27.65^***^ | [12.88,42.42] | -0.00 | [-0.02,0.02] | 0.00 | [-0.02,0.02] |
| 0.OccAct | 0.00 | [0.00,0.00] | 0.00 | [0.00,0.00] | 0.00 | [0.00,0.00] | 0.00 | [0.00,0.00] | 0.00 | [0.00,0.00] | 0.00 | [0.00,0.00] |
| 1.OccAct | -1.49^***^ | [-2.04,-0.94] | 1.58^***^ | [1.26,1.89] | 1.82^***^ | [1.49,2.15] | 0.21 | [-5.57,5.98] | 0.04^***^ | [0.03,0.05] | 0.01^***^ | [0.01,0.02] |
| 2.OccAct | -1.37^***^ | [-1.89,-0.86] | 1.57^***^ | [1.28,1.87] | 2.26^***^ | [1.95,2.57] | 3.79 | [-1.66,9.25] | 0.05^***^ | [0.04,0.05] | 0.02^***^ | [0.01,0.03] |
| 3.OccAct | -0.99^*^ | [-1.96,-0.02] | 1.93^***^ | [1.38,2.49] | 3.21^***^ | [2.63,3.79] | 6.62 | [-3.68,16.91] | 0.06^***^ | [0.05,0.07] | 0.03^***^ | [0.01,0.04] |
| 0.Smoking | 0.00 | [0.00,0.00] | 0.00 | [0.00,0.00] | 0.00 | [0.00,0.00] | 0.00 | [0.00,0.00] | 0.00 | [0.00,0.00] | 0.00 | [0.00,0.00] |
| 1.Smoking | 0.49^*^ | [0.07,0.91] | -0.37^**^ | [-0.61,-0.13] | -0.16 | [-0.41,0.09] | -4.81^*^ | [-9.24,-0.38] | -0.00 | [-0.01,0.00] | -0.00 | [-0.01,0.00] |
| 2.Smoking | -0.68 | [-1.58,0.22] | -0.02 | [-0.53,0.50] | -0.06 | [-0.60,0.48] | -6.56 | [-16.10,2.98] | -0.00 | [-0.02,0.01] | 0.00 | [-0.01,0.01] |
| 3.Smoking | -0.27 | [-0.91,0.37] | -0.02 | [-0.39,0.34] | 0.03 | [-0.35,0.42] | -6.73 | [-13.51,0.05] | -0.01^**^ | [-0.02,-0.00] | -0.01^*^ | [-0.02,-0.00] |
| wake_time_h | -0.29^**^ | [-0.50,-0.08] | 0.04 | [-0.08,0.16] | 0.02 | [-0.10,0.15] | -7.47^***^ | [-9.71,-5.24] | 0.02^***^ | [0.02,0.02] | 0.00 | [-0.00,0.01] |
| daily_n_stps | 0.00^***^ | [0.00,0.00] | 0.00^***^ | [0.00,0.00] | 0.00^***^ | [0.00,0.00] | 0.02^***^ | [0.02,0.02] | 0.00^**^ | [0.00,0.00] | 0.00^***^ | [0.00,0.00] |
| _cons | 34.79^***^ | [31.33,38.25] | 3.76^***^ | [1.78,5.74] | 3.56^***^ | [1.49,5.63] | 129.27^***^ | [92.67,165.87] | -0.06^*^ | [-0.11,-0.01] | 0.18^***^ | [0.13,0.23] |
| p | 0.00 |  | 0.00 |  | 0.00 |  | 0.00 |  | 0.00 |  | 0.00 |  |
| 95% confidence intervals in brackets  ^*^ *p* < 0.05, ^**^ *p* < 0.01, ^***^ *p* < 0.001 | | | | | | | | | | | | |
